# Population pharmacokinetics and pharmacodynamics of a novel vascular adhesion protein-1 inhibitor using a multiple-target mediated drug disposition model

**DOI:** 10.1007/s10928-020-09717-w

**Published:** 2020-09-15

**Authors:** Nelleke Snelder, Sven Hoefman, Alberto Garcia-Hernandez, Hartmut Onkels, Tobias E. Larsson, Kirsten R. Bergmann

**Affiliations:** 1 LAP&P Consultants BV, Archimedesweg 31, 2333 CM Leiden, The Netherlands; 2 Astellas Pharma Europe BV, Global Development, Sylviusweg 62, 2333 BE Leiden, The Netherlands

**Keywords:** VAP-1 inhibition, Albuminuria, Diabetic kidney disease, Population pharmacokinetic modeling

## Abstract

ASP8232 is a novel inhibitor of vascular adhesion protein-1 that was under evaluation for reducing residual albuminuria in patients with diabetic kidney disease. To characterize the pharmacokinetics (PK) of ASP8232 and its effect on vascular adhesion protein 1 (VAP-1) plasma activity and VAP-1 concentrations (pharmacodynamics, PD) in an integrated and quantitative manner, a target mediated drug disposition model was developed based on pooled data from four completed clinical trials with ASP8232 in healthy volunteers, and in patients with diabetic kidney disease and diabetic macular edema, respectively. In this model, the binding of ASP8232 to its soluble and membrane-bound target in the central and peripheral compartments were included. The model was able to adequately describe the non-linear PK and PD of ASP8232. The observed difference in PK between healthy volunteers and renally impaired patients could be explained by an effect of baseline estimated glomerular filtration rate on ASP8232 clearance and relative bioavailability. The relationship between ASP8232 concentration and VAP-1 inhibition was successfully established and can be applied to simulate drug exposure and degree of VAP-1 inhibition for any given dose of ASP8232 across the spectrum of renal function.

**Electronic supplementary material:**

The online version of this article (10.1007/s10928-020-09717-w) contains supplementary material, which is available to authorized users.

## Introduction

Vascular adhesion protein-1 (VAP-1) is a 180 kDa transmembrane homodimeric glycoprotein [1, 2]. As an amine oxidase, it catalyzes the oxidation of amines to form aldehydes, hydrogen peroxide and ammonia. VAP-1 has both enzymatic and non-enzymatic activities, and plays an important role in leukocyte trafficking and adhesion [3–5]. Membrane bound VAP-1 (mVAP-1) can be cleaved by metalloproteinases and shed as soluble VAP-1 (sVAP-1). sVAP-1 concentrations increase at sites of inflammation and its upregulation and subsequent increased oxidase activity plays a role in many inflammatory diseases [6, 7]. sVAP-1 is upregulated in diabetic patients [8, 9] and subjects with early stages of chronic kidney disease [10], where VAP-1 activity is thought to play an important pathogenic role.

ASP8232 is a small molecule VAP-1 inhibitor that was evaluated for the treatment of diabetic kidney disease (DKD) as an add-on to first-line antihypertensive therapy in a Phase 2 study (ALBUM study). In this trial, ASP8232 was found effective in reducing albuminuria following a 12-week treatment with daily oral doses of 40 mg ASP8232 in DKD patients receiving standard of care, i.e. angiotensin converting enzyme inhibitor (ACEi) or angiotensin receptor blocker (ARB) therapy [11]. A 98.6% inhibition of VAP-1 activity was observed after 2 weeks of ASP8232 treatment, and this suppression remained throughout the treatment duration [11]. However, a quantitative understanding of the pharmacokinetic-pharmacodynamic (PK-PD) relationship following ASP8232 administration is currently lacking. Characterizing this relationship would provide the ability to predict the time to steady-state ASP8232 concentration and the VAP-1 inhibition response to different ASP8232 concentrations, to identify individual covariate effects which influence this relationship, and to simulate the expected dose – PD response curve for DKD patients.

Population PK-PD models describe the absorption and disposition of a drug and characterize the relationship between compound and its target. Occasionally, the target can influence the disposition, i.e. distribution and elimination processes, of the drug. These processes can be described using target mediated drug disposition (TMDD) models, which incorporate target binding and turnover concepts and allow for a semi-mechanistic interpretation of drug disposition [12, 13]. Estimating the parameters for a full TMDD model requires a rich dataset and information during each phase of the PK profile [13]. Therefore, approximations to the full TMDD model were introduced, such as the quasi-equilibrium, quasi-steady state and Michaelis–Menten TMDD approximations [14–16]. These approximations rely on certain assumptions, e.g. rapid binding of drug to its target, which need to be checked for their validity given the data.

In this study, a population TMDD model was developed which characterizes the PK-PD relationship of ASP8232 and quantifies the relationship between VAP-1 concentrations and activity. The model simultaneously describes PK, sVAP-1 and VAP-1 plasma activity data from four clinical trials over a range of oral APS8232 doses in healthy volunteers, DKD patients and patients with diabetic macular edema (DME). The model was applied to predict the time to steady-state ASP8232 concentration and expected VAP-1 inhibition following a one year treatment with different ASP8232 doses.

## Methods

### Clinical studies

ASP8232 and sVAP-1 plasma concentrations, and VAP-1 plasma activity measurements from four clinical studies were included in the PK-PD analysis:

1. A Phase 1, first-in-human study in healthy male and female subjects (study 8232-CL-0001),
2. a Phase 1 study in patients with renal impairment and patients having Type 2 diabetes mellitus (T2DM) with chronic kidney disease (CKD) (study 8232-CL-0002),
3. a Phase 2 study in patients with DME (VIDI study),
4. a Phase 2 study in patients with DKD (ALBUM study; [11]).

sVAP-1 concentrations were not measured in studies 8232-CL-0001 and 8232-CL-0002. In total, 3498 ASP8232 plasma concentration records, 5893 VAP-1 plasma activity records and 1714 VAP-1 plasma concentration records from these four clinical studies were included in the PK-PD analysis. A study overview and the distributions of continuous covariates and baseline PD measurements and categorical covariates can be found in Table 1, 2 and 3, respectively.

**Table 1:** Study overview and number of records used for PK-PD modeling

Study	Population	Subjects (n)	Males (%)	ASP8232 conc. (n)	VAP-1 activity (n)	VAP-1 conc. (n)	ASP8232 dose range (loading dose)
8232-CL-0001	Healthy	92^a^	80.4	1677	3082	–	0.1–100 mg single dose^a^ 0.2–200 mg QD^b^ (1–600 mg)
8232-CL-0002	RI^c^ / DKD	55	63.6	1070	1134	–	200 mg single dose /150 mg QD (250 mg)
VIDI study	DME	96	50.0	366	706	709	40 mg QD
ALBUM study	DKD	120	77.5	385	971	1005	40 mg QD
Pooled data	All of above	363	68.9	3498	5893	1714	All of above

^a^Single doses of 300–1000 mg (24 subjects) and multiple dose of 800 mg (12 subjects) were not included in analysis

^b^Once daily

^c^Renally Impaired

**Table 2:** Baseline and continuous covariate distributions

Variable (unit)	Study	N	Min	5th %	Median	95th %	Max
Body weight (kg)	Pooled data	363	49.1	58.1	83.6	123	158
	8232-CL-0001	92	49.1	55.4	73.6	93.8	102
	8232-CL-0002	55	55.9	56.7	81.1	96.5	101
	VIDI study	96	53.2	64.0	87.3	135	158
	ALBUM study	120	53.5	65.9	91.7	127	153
eGFR	Pooled data	363	14.2	27.0	64.0	117	136
(mL/min/1.73m^2^)	8232-CL-0001	92	73.7	80.4	104	127	136
	8232-CL-0002	55	14.2	19.3	55.8	107	113
	VIDI study	96	17.5	29.4	73.4	103	110
	ALBUM study	120	22.1	26.9	44.0	63.4	71.5
Baseline VAP-1	Pooled data	358	81.1	1981	3930	5494	7030
plasma activity (nM)	8232-CL-0001	92	1334	2358	3946	5146	6351
	8232-CL-0002	55	2122	2646	4001	6033	6782
	VIDI study	96	106	2272	4030	5768	6310
	ALBUM study	115	81.1	1863	3820	5270	7030
Baseline VAP-1	Pooled data	211	1.87	3.26	5.93	10.5	13.9
plasma concentration	VIDI study	96	2.28	3.18	5.74	9.73	13.9
(pmol/mL/h)	ALBUM study	115	1.87	3.33	6.20	10.9	12.1

**Table 3:** Categorical covariate distribution

Variable (unit)	Study	N	Male (%)
Sex	Pooled data	363	68.9
	8232-CL-0001	92	80.4
	8232-CL-0002	55	63.6
	VIDI study	96	50
	ALBUM study	120	77.5

Study 8232-CL-0001 was a Phase 1, double-blind, randomized, placebo-controlled, single and multiple ascending oral dose study of ASP8232 in healthy subjects (unpublished data). In the single dose cohort, 10 doses were evaluated (0.1, 1, 3, 10, 30, 100, 300, 1000, 3000 or 6000 mg) with 8 subjects per group (6 active; 2 placebo). The multiple dose cohort of the study consisted of 3 groups, each with 16 subjects (12 active; 4 placebo). In the low dose group, a single dose of 0.2 mg ASP8232 or placebo was administered on day 1, followed by a loading dose of 1 mg on day 8, and thereafter 13 daily doses of 0.2 mg until day 21. In the mid dose group, subjects received 200 mg ASP8232 on day 1, 600 mg (loading dose) on day 2, and 200 mg daily from day 3 to 14, or placebo from day 1–14. In the high dose group, subjects received 800 mg ASP8232 on day 1, 1100 mg (loading dose) on day 2, and 800 mg daily from day 3 to 14, or placebo from day 1–14.

Study 8232-CL-0002 was a Phase 1 study to evaluate the effect of renal impairment on the PK, PD and safety of ASP8232 (part 1) and a multiple dose, placebo-controlled study in subjects with Type 2 diabetes mellitus and CKD, i.e. DKD (part 2) (ClinicalTrials.gov NCT02218099). In part 1, 8 subjects with either mild, moderate or severe renal impairment and 16 matching healthy subjects received a single 200 mg oral ASP8232 dose. In part 2, 15 DKD subjects (10 active; 5 placebo) received a loading dose of 250 mg ASP8232 on day 1 followed by daily dosing of 150 mg ASP8232 for 27 days.

Study 8232-CL-3001 (VIDI study) was a Phase 2, double-blind, randomized study in subjects with DME (ClinicalTrials.gov NCT02302079). Data from a 4-week screening, 12-week treatment and 12-week follow-up period were available for 31 subjects receiving placebo and 0.3 mg intravitreal ranibizumab injections, 32 subjects receiving 40 mg daily oral ASP8232 and 33 subjects receiving 40 mg daily oral ASP8232 and 0.3 mg intravitreal ranibizumab injections.

Study 8232-CL-0004 (ALBUM study; [11]) was a Phase 2 double-blind, randomized, placebo-controlled study in DKD patients (ClinicalTrials.gov NCT02358096). Data from a 1-week screening, 5-week pre-treatment, 12-week treatment and 24-week follow-up period were available for 60 subjects receiving 40 mg daily oral ASP8232 and 60 subjects receiving placebo.

### Analytical methods

Plasma samples for the PK of ASP8232 were analyzed using a liquid chromatography-mass spectrometry assay measuring total ASP8232 concentrations, i.e. ASP8232 unbound and bound to sVAP-1 in plasma. sVAP-1 plasma concentrations were analyzed using an enzyme linked immunosorbent assay based on a commercial kit (Human sVAP-1 ELISA, BE59091, IBL International, Hamburg, Germany; with assay performance according to the packaging insert of the manufacturer) measuring total sVAP-1 concentrations, i.e. sVAP-1 unbound and bound to ASP8232 in plasma. Plasma samples for enzymatic activity of VAP-1 were analyzed using an enzymatic assay with a radioactive substrate (14C-benzylamine hydrochloride) via liquid–liquid extraction followed by liquid scintillation counting. These assays were formally validated and the lower limit of quantification was 0.1 ng/mL and 0.625 ng/mL for the plasma PK and sVAP-1 assay, respectively. For plasma VAP-1 activity, no measurements were reported below the quantification limit.

### Main modeling assumptions

The following assumptions, relevant for the modeling analysis, were made:

1. The PK of ASP8232 was not influenced by binding to any other target than VAP-1. ASP8232 could potentially bind to sVAP-1 in the central compartment and to mVAP-1 in the central and peripheral compartments.
2. The measured VAP-1 plasma activity was driven by the unbound sVAP-1 plasma concentrations and this relationship was assumed to be constant over time.
3. Compared with other processes, binding of ASP8232 to VAP-1 was assumed to be rapid while VAP-1 turnover and elimination of the VAP-1-ASP8232 complex were assumed to be negligible.
4. The dissociation constant (K_D_) was assumed to be the same for binding of ASP8232 to sVAP-1 and mVAP-1 across all model compartments.
5. The VAP-1 concentration may differ for each model compartment.
6. The molecular weight, used to convert dose or concentrations to molar units, was 444 g/mol for ASP8232 (free base) and 84,622 g/mol for the VAP-1 monomer (UniProt nr. Q16853) with difference between mVAP-1 and sVAP-1 assumed to be negligible as the cleavage site for mVAP-1 is close to the membrane [7].

### Population analysis methodology and computation

The PK-PD data were analyzed using a population approach, also called mixed-effects modeling. A population model is composed of a structural model parameterized with structural (fixed effects) parameters, and a stochastic model quantifying the inter-individual (IIV) and residual variability [17]. Parameter estimation and model simulations were performed using NONMEM version 7.3 in combination with PsN version 4.6.0 [18, 19]. Processing of NONMEM output was performed using R version 3.3.2 and RStudio version 1.0.44.

### ASP8232 PK-PD TMDD model development

The starting PK-PD TMDD model was a first-order absorption three compartmental model with binding of ASP8232 to VAP-1 according to the main modeling assumptions. In short, target binding was assumed to be at equilibrium and consequently the dissociation constant (K_D_) was given by Eq. 1.

1 $$ K_{D} = \frac{{\left[ {ASP8232_{unbound} } \right] \cdot \left[ {VAP1_{unbound} } \right]}}{{\left[ {ASP8232 - VAP1complex} \right]}} $$

From this equation the free fraction was derived for (i) ASP8232 in the central compartment (ϕ_drug,c_), (ii) ASP8232 in each peripheral compartment ( ϕ_drug,p1_ and ϕ_drug,p2_) and (iii) total VAP1 (tVAP1_c_ = sVAP1_c_ + mVAP1_c_) in the central compartment (ϕ_tVAP1,c_) (Eq. 2–5).

2 $$ \phi_{drug,c} = \frac{{\left[ {ASP8232_{c} } \right] - \left[ {tVAP1_{c} } \right] - K_{D} + \sqrt {(\left[ {ASP8232_{c} } \right] - \left[ {tVAP1_{c} } \right] - K_{D} )^{2} + 4 \cdot K_{D} \cdot \left[ {ASP8232_{c} } \right]} }}{{2 \cdot [ASP8232_{c} ]}} $$

3 $$ \phi_{drug,p1} = \frac{{\left[ {ASP8232_{p1} } \right] - \left[ {mVAP1_{p1} } \right] - K_{D} + \sqrt {(\left[ {ASP8232_{p1} } \right] - \left[ {mVAP1_{p1} } \right] - K_{D} )^{2} + 4 \cdot K_{D} \cdot \left[ {ASP8232_{p1} } \right]} }}{{2 \cdot [ASP8232_{p1} ]}} $$

4 $$ \phi_{drug,p2} = \frac{{\left[ {ASP8232_{p2} } \right] - \left[ {mVAP1_{p2} } \right] - K_{D} + \sqrt {(\left[ {ASP8232_{p2} } \right] - \left[ {mVAP1_{p2} } \right] - K_{D} )^{2} + 4 \cdot K_{D} \cdot \left[ {ASP8232_{p2} } \right]} }}{{2 \cdot [ASP8232_{p2} ]}} $$

5 $$ \phi_{tVAP1,c} = \frac{{\left[ {tVAP1_{c} } \right] - \left[ {ASP8232_{c} } \right] - K_{D} + \sqrt {(\left[ {tVAP1_{c} } \right] - \left[ {ASP8232_{c} } \right] - K_{D} )^{2} + 4 \cdot K_{D} \cdot \left[ {tVAP1_{c} } \right]} }}{{2 \cdot \left[ {tVAP1_{c} } \right]}} $$

With the derived free fractions, the starting TMDD PK-PD model was described by the following differential equations (Eq. 6–9) with A1, A2, A3 and A4 defined as the drug amount in the depot, central, first peripheral and second peripheral compartment, respectively.

6 $$ \frac{{dA_{1} }}{dt} = - k_{a} \cdot A_{1} $$

7 $$ \frac{{dA_{2} }}{dt} = k_{a} \cdot A_{1} - \phi_{drug,c} \cdot k_{20} \cdot A_{2} - \phi_{drug,c} \cdot k_{23} \cdot A_{2} + \phi_{drug,p1} \cdot k_{32} \cdot A_{3} - \phi_{drug,c} \cdot k_{24} \cdot A_{2} + \phi_{drug,p2} \cdot k_{42} \cdot A_{4} $$

8 $$ \frac{{dA_{3} }}{dt} = \phi_{drug,c} \cdot k_{23} \cdot A_{2} - \phi_{drug,p1} \cdot k_{32} \cdot A_{3} $$

9 $$ \frac{{dA_{4} }}{dt} = \phi_{drug,c} \cdot k_{24} \cdot A_{2} - \phi_{drug,p2} \cdot k_{42} \cdot A_{4} $$

The dependent variables for the PK-PD analysis were the log-transformed total ASP8232 and sVAP-1 plasma concentrations, as well as VAP-1 plasma activity, which were predicted by the model according to the following individual prediction (IPRED) Eqs. (10–12).

10 $$ IPRED_{drug} = \log \left( {\phi_{drug,c} \cdot \frac{{A_{2} }}{V2} + \left( {1 - \phi_{drug,c} } \right) \cdot \frac{{A_{2} }}{V2} \cdot \frac{{sVAP1_{c} }}{{tVAP1_{c} }}} \right) $$

11 $$ IPRED_{sVAP1} = \log \left( {sVAP1_{c} } \right) $$

12 $$ IPRED_{{VAP1_{act} }} = \log \left( {SL \cdot \left( {\phi_{tVAP1,c} .sVAP1_{c} } \right)^{POW} } \right) $$

Log-transformed data were modeled with additive residual error. Initially, improvements or simplifications of the structural model were considered, which included the estimation of one shared volume parameter for the two peripheral compartments. Absorption transit compartments were evaluated as a mechanism to account for a delay in oral absorption. Alternative relationships between sVAP-1 plasma concentrations and VAP-1 plasma activity, such as a linear relationship, were evaluated.

Hereafter, an appropriate individual random effect structure was implemented following standard model selection and acceptance criteria (see section Model Evaluation). Inter-individual variability (IIV) was included by using an exponential relationship assuming log-normal distributions. Subsequently, a limited covariate analysis was performed to allow integration of data from the different studies. Since an exhaustive covariate analysis was not a specific objective of this analysis, only key covariates that were expected to explain differences between studies were selected, e.g. because of different inclusion criteria. The possible effect of sex, body weight and baseline estimated glomerular filtration rate (eGFR) using the CKD-EPI equation [20] were evaluated on the model parameters with IIV included. In addition, these possible covariate effects were also evaluated on the volume of distribution as it is expected that body weight and sex may have an effect on this parameter based on physiological grounds. A power or sigmoid emax relationship was evaluated for continuous covariates, while a sex effect was tested using a separate estimate for females. The possible effect of each covariate was tested in a univariate manner and ranked according to the extent of the drop in objective function value. Subsequently, each covariate that had a significant effect at p < 0.01 (drop of at least 6.63 points; χ^2^, 1 degree of freedom) was added to the model one-by-one according to their initial rank order until no more covariates could be added, i.e. p > 0.01. Each relationship was retained if upon backward deletion, it was significant at a stricter criterion of p < 0.001 (drop of at least 10.8 points; χ^2^, 1 degree of freedom).

### Model evaluation

Standard model acceptance criteria were applied during model development and a range of goodness-of-fit plots were inspected visually to evaluate the model fit [21–24]. Visual predictive checks (VPC [25, 26]) were performed based on 100 replications of the original dataset, using scheduled time as independent variable and stratified per variable, treatment group and study.

For evaluation purposes, VAP-1 activity values were transformed to VAP-1 inhibition percentages via the individual baseline VAP-1 activity as follows (Eq. 13):

13 $$ VAP1_{inhibition,\% } = 100 \cdot \frac{{VAP1_{act,bsl,i} - VAP1_{act,j,i} }}{{VAP1_{act,bsl,i} }} $$

where VAP1_act,bsl,i_ is the median of pre-dose VAP-1 activity observations for an individual i, and VAP1_act,j,i_ the jth VAP-1 activity observation of individual i.

### Model simulation

Individual parameters were obtained using empirical Bayes estimation (EBE) in NONMEM. Based on these parameters and the final model, simulations were performed for a virtual DKD population predicting the AUC_24h,52w_ and VAP-1_inhibition,52w_ for a dose range of 0.1 to 40 mg daily dosing, which have potential to be used in a dose-finding study. These simulations were based on individual predictions (IIV included; parameter uncertainty and residual variability excluded) based on 10,000 virtual DKD patients, with sex distribution as observed in the ALBUM study and individual eGFR values sampled from a normal distribution with mean and standard deviation as observed in the ALBUM study. In addition, simulations were performed to predict the ASP8232 concentration—time profile after 52 weeks of daily ASP8232 dosing for a typical male subject with eGFR of 44 mL/min/1.73m^2^, i.e. the median value observed in the ALBUM study. The 52 week dosing period was selected to ensure steady state was reached at all dose levels. From these simulations, the following key secondary ASP8232 PK parameters were derived: steady-state exposure (AUC_24h,52w_), maximum plasma concentration at steady state (Cmax_52w_) and the corresponding time at which Cmax is reached (Tmax_52w_), the time to reach steady state (T_ss_) and the apparent half-life (t1/2). T_ss_ was defined as the time to reach 97% of the trough concentration predicted after 52 weeks of daily ASP8232 dosing. The apparent half-life (t1/2) was derived from the simulated T_ss_ by dividing the simulated T_ss_ by 5.

## Results

### ASP8232 PK-PD TMDD model

**Fig. 1:**
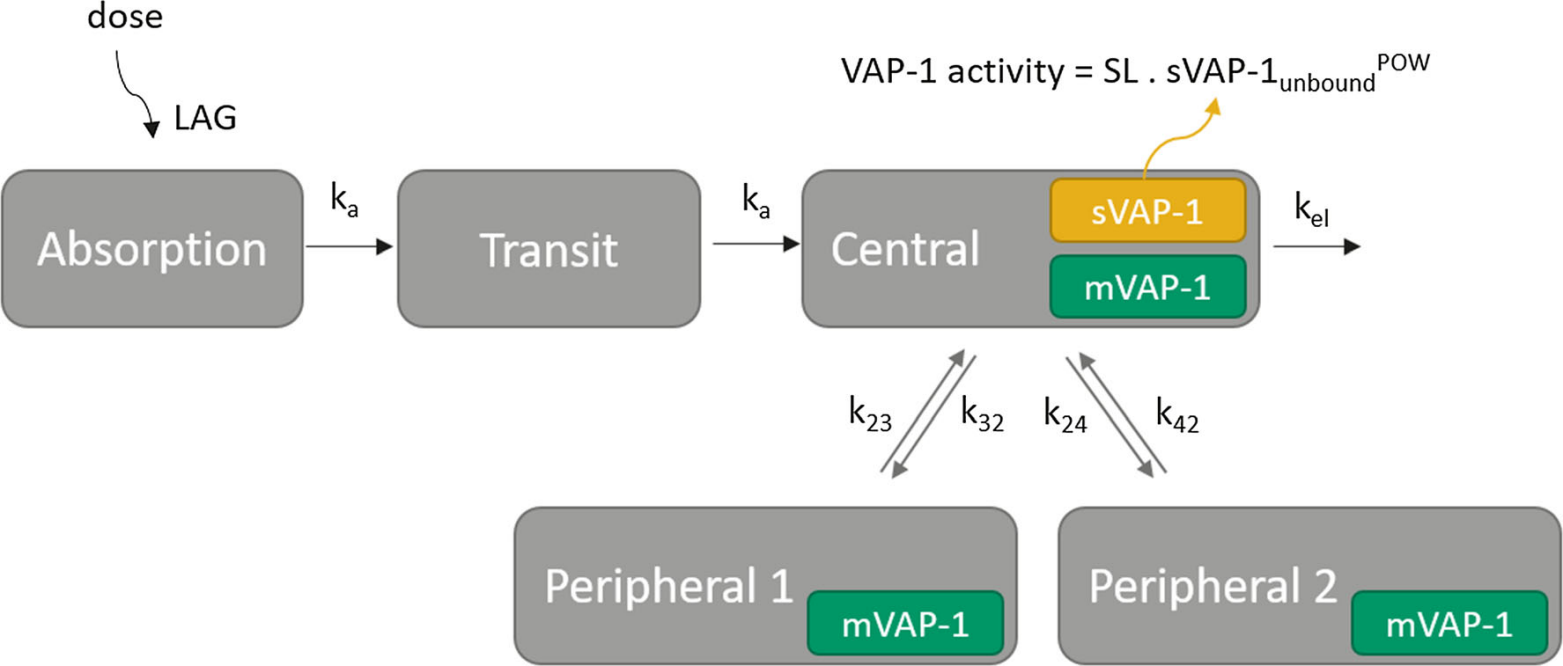
Schematic representation of the ASP8232 PK-PD model. Drug absorption is characterized via estimated lag time and ka including one transit compartment (Transit). Unbound ASP8232 is eliminated from the central compartment via kel, and can distribute to the peripheral compartments. In the central compartment ASP8232 can bind to sVAP-1_c_ or mVAP-1_c_, while in the peripheral compartments ASP8232 can bind to mVAP-1_p1_ = mVAP-1_p2_. ASP8232 in complex with target cannot distribute or be eliminated, and binding is assumed to occur at equilibrium with estimated K_D_. Rate constants kel, k23, k32, k24 and k42 are secondary parameters, based on estimated parameters CL, V2, V3 = V2, V4, Q and Q2. VAP-1 plasma activity is a function of free soluble VAP-1 plasma concentration according to a power model with estimated slope and power parameters, SL and POW, respectively

The PK-PD of ASP8232 was best described by a three compartmental model with ASP8232 distribution from the central compartment (parameterized with volume V2) into two peripheral compartments (with volume V3 = V2 to reduce parameter correlations, and inter-compartmental clearance Q, and volume V4 and intercompartmental clearance Q2, respectively) (Fig. 1). Elimination of unbound ASP8232 plasma concentration from the central compartment was characterized by estimating clearance (CL), and thus the elimination rate constant (k_el_) via CL/V2. Drug absorption was modeled via a first order absorption rate constant (k_a_) and the addition of a transit compartment with estimated lag time (LAG). The addition of the transit compartment resulted in a more stable model with similar objective function value (difference of 0.0640 points). The concentration of sVAP-1 in the central compartment (sVAP-1_c_) was estimated. The concentrations (amount accessible from the central or peripheral compartment volumes) of mVAP-1 in the central and two peripheral compartments (mVAP1_c_, mVAP1_p1_ and mVAP1_p2_) were estimated relative to sVAP-1_c_. Binding of ASP8232 to sVAP-1_c_ and to mVAP-1 in all compartments was parameterized with K_D_. VAP-1 plasma activity was a function of unbound sVAP-1 plasma concentration according to a power model with estimated slope parameter (SL) and power coefficient (POW). IIV was implemented on CL, SL and sVAP-1_c_. Data were log-transformed and modeled with additive residual error, with separate estimates for data from the Phase 2 studies (ALBUM and VIDI study) implemented by estimating a factor for ASP8232 plasma concentrations and VAP-1 plasma activities. In addition to estimation of the variances for IIV on CL, sVAP-1_c_ and SL, all covariances were estimated (full omega block). IIV was not included on V2, due to instability of the resulting models.

**Fig. 2:**
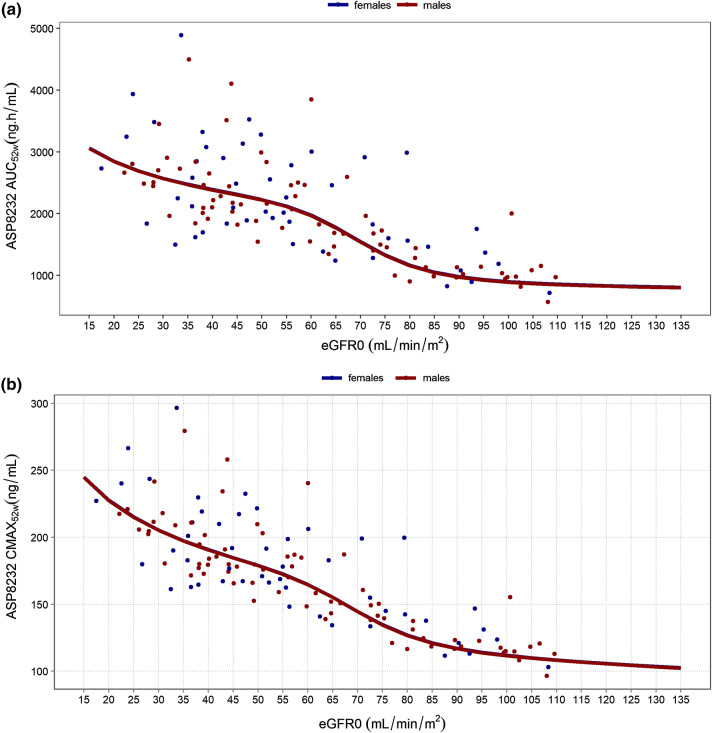
Influence of sex and eGFR on ASP8232 AUC_24h,52w_ (**a**) and Cmax_52w_ (**b**) based on empirical bayes estimates from the model for subjects receiving 40 mg daily dosing (ALBUM and VIDI study). Individual (dots) and population predictions (lines) for females (blue) and males (red). The line for females is barely visible due to overlap with males (Color figure online)

The final model contained an effect of eGFR on CL, which was implemented via a sigmoid Emax relationship with power estimate fixed to a large value (10) indicating an on or off effect relative to the estimated EC_50_ of this relationship. In addition, a sex effect on the VAP-1 concentrations, and an effect of eGFR on the relative bioavailability via a power relationship, were included in the model. The VAP-1 concentrations were found to be 12.5% higher for females. The combined effect of all covariates on ASP8232 steady-state exposure, AUC_24h,52w_, and maximum steady-state concentration, Cmax_52w_, is presented in Fig. 2. AUC_24h,52w_ and Cmax_52w_ are expected to increase with decreasing eGFR, i.e. with impaired renal function. The EBE-based individual derived AUC_24h,52w_ and Cmax_52w_ are scattered around the population prediction for this relationship (Fig. 2; dots). The influence of sex on both parameters is predicted to be minimal, as seen by the overlapping lines for a typical male and female subject (Fig. 2; lines). During model development, it was found that differences in exposure between healthy volunteers and patients were covered entirely by their difference in eGFR.

The ASP8232 exposure appeared to increase less than proportional for doses ≥ 300 mg. These dose levels were excluded during model development, as they were outside the considered clinically relevant exposure range. During development, outliers were identified (defined as CWRES > 3 or < -3) and excluded from further analysis to stabilize the model. As such, 1.8% of the ASP8232 plasma concentrations, 2.3% of the VAP-1 plasma activities and 1.4% of the VAP-1 plasma concentrations were identified as outliers. Re-estimation of the model including all data did not noticeably impact the estimates or any conclusions drawn.

### Model evaluation

**Fig. 3:**
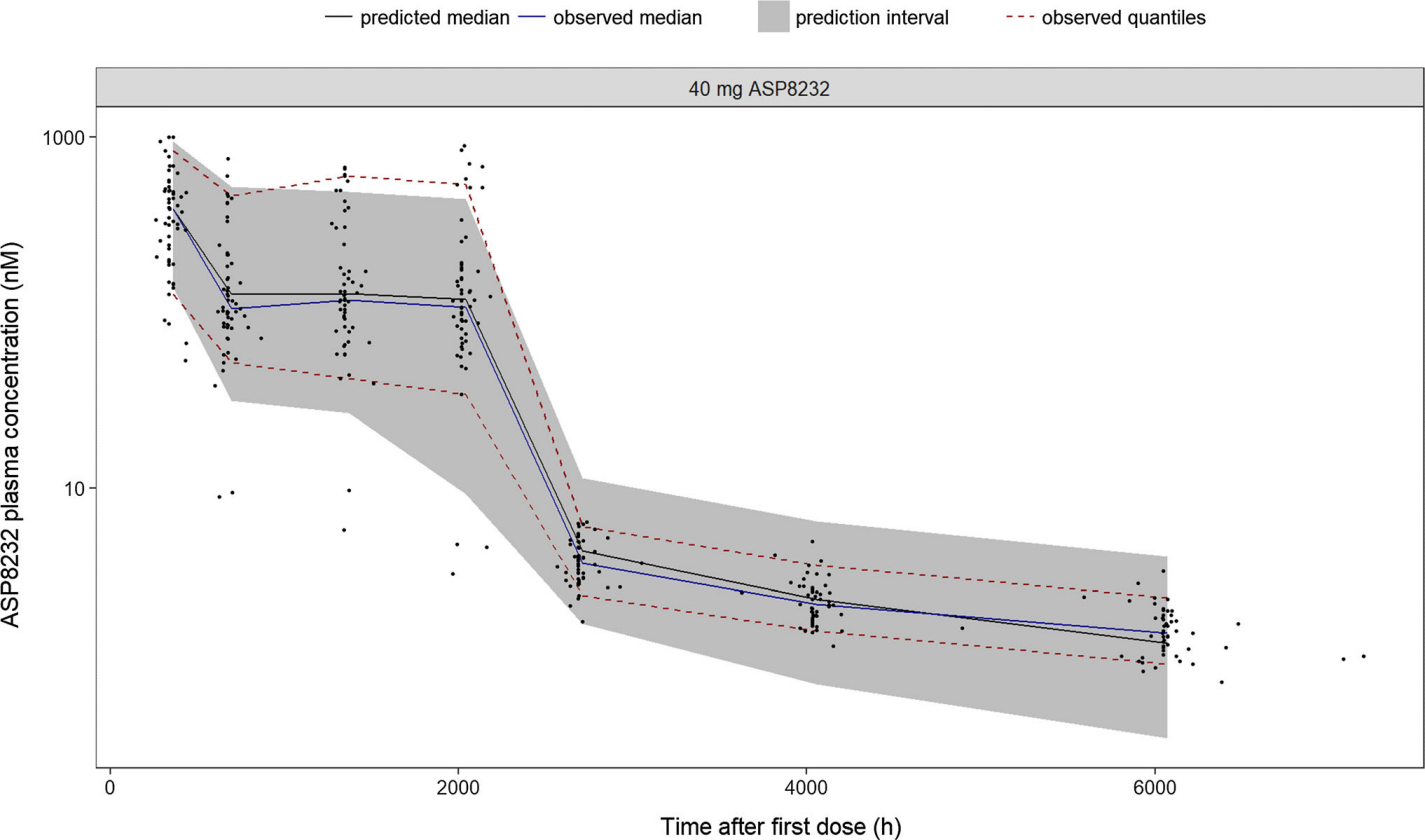
Visual predictive check of ASP8232 plasma concentration in the ALBUM study. ASP8232 was dosed once daily during 12 weeks (2016 h). ASP8232 plasma concentration were measured at week 2 (336 h), 4 (672 h), 8 (1344 h), 12 (2016 h), 16 (2688 h), 24 (4032 h) and 36 (6048 h). Observed data (black dots), observed median (blue line), observed 5th and 95th percentiles (dashed red lines), predicted median (black line) and 90% prediction interval (shaded area) covering the predicted 5th and 95th percentiles (Color figure online)

**Fig. 4:**
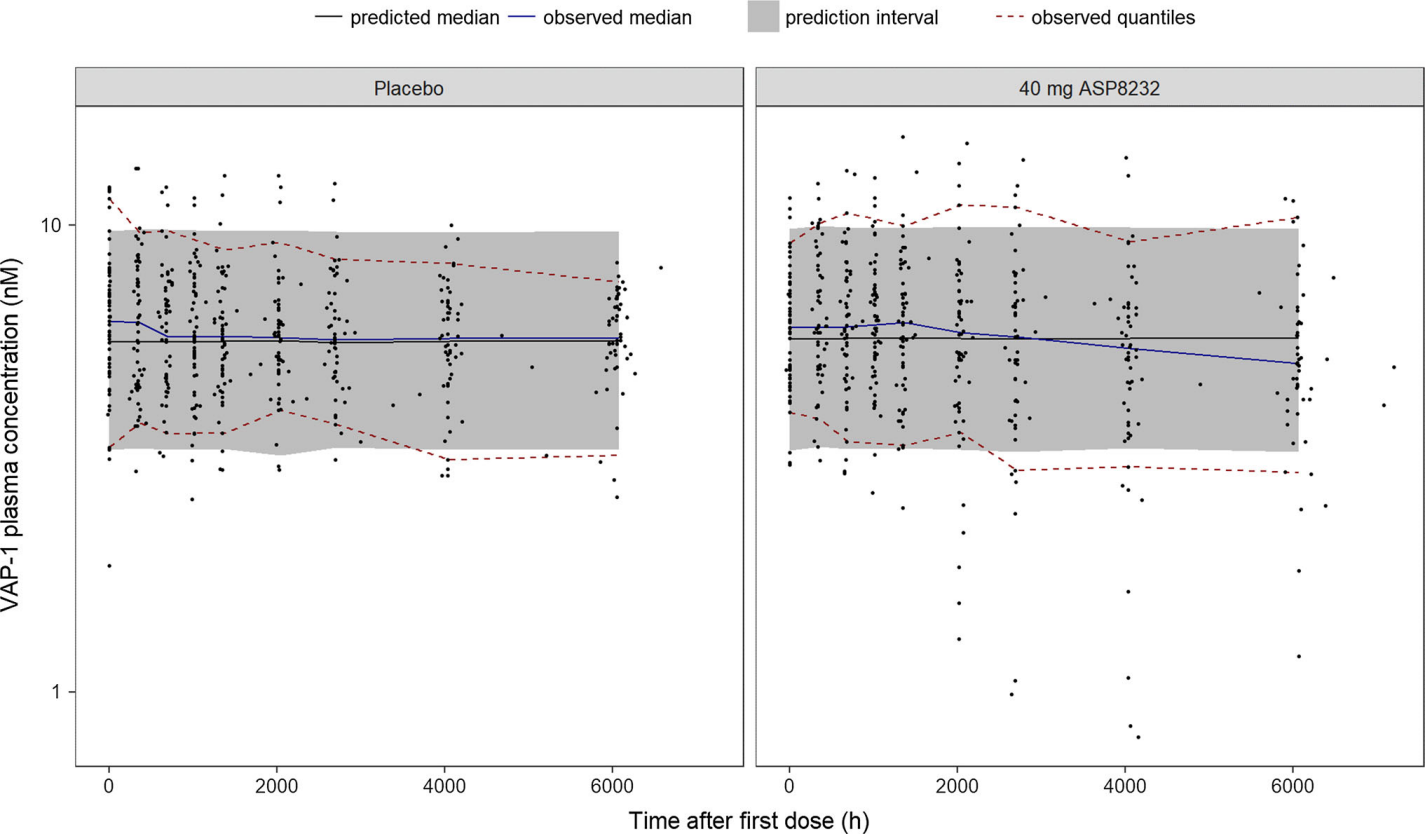
Visual predictive check of VAP-1 plasma concentration in the ALBUM study. ASP8232 or placebo was dosed once daily during 12 weeks (2016 h). VAP-1 plasma concentration was measured at baseline (0 h) and at week 2 (336 h), 4 (672 h), 6 (1008 h), 8 (1344 h), 12 (2016 h), 16 (2688 h), 24 (4032 h) and 36 (6048 h). Observed data (black dots), observed median (blue line), observed 5th and 95th percentiles (dashed red lines), predicted median (black line) and 90% prediction interval (shaded area) covering the predicted 5th and 95th percentiles (Color figure online)

**Fig. 5:**
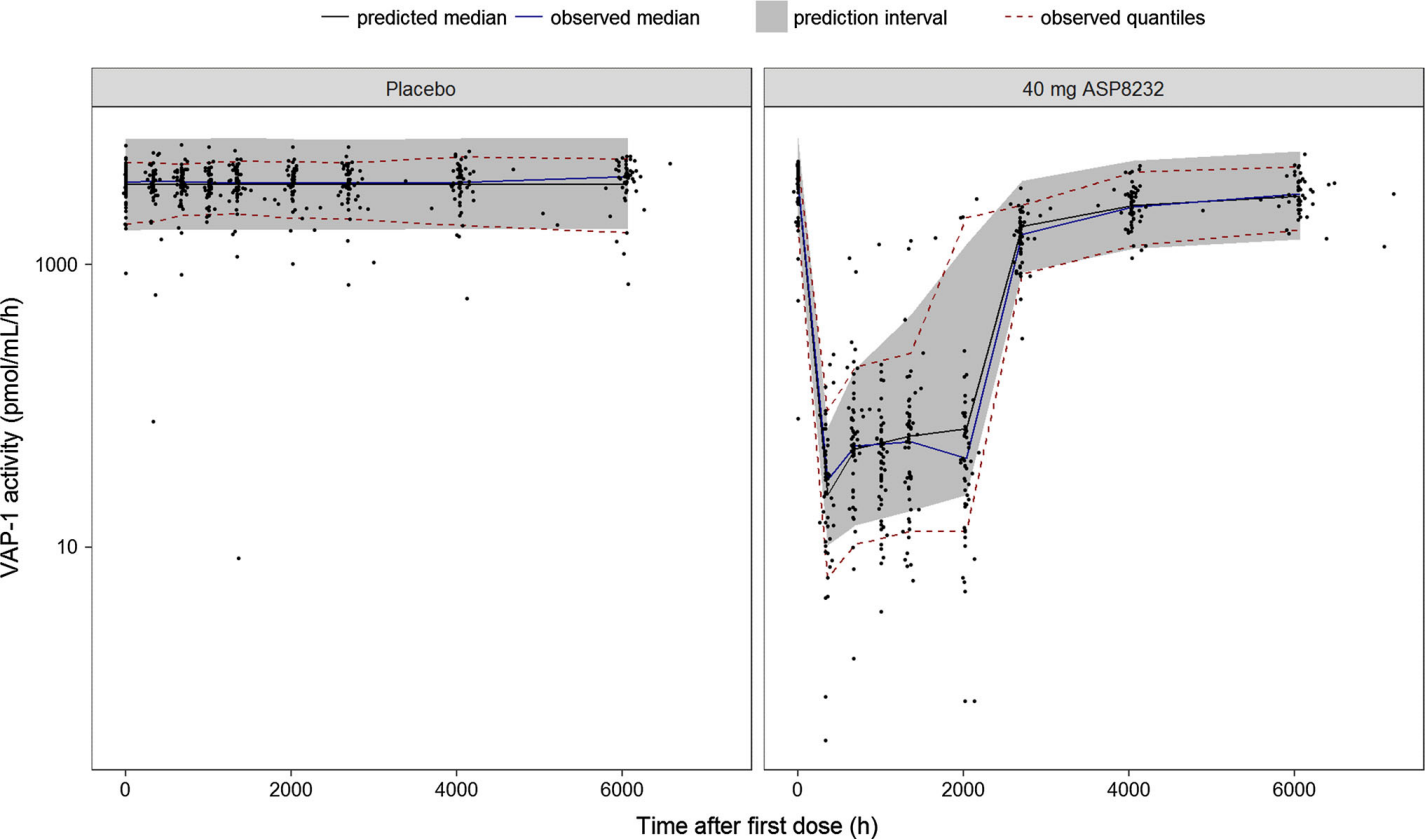
Visual predictive check of VAP-1 plasma activity in the ALBUM study. ASP8232 or placebo was dosed once daily during 12 weeks (2016 h). VAP-1 plasma activity was measured at baseline (0 h) and at week 2 (336 h), 4 (672 h), 6 (1008 h), 8 (1344 h), 12 (2016 h), 16 (2688 h), 24 (4032 h) and 36 (6048 h). Observed data (black dots), observed median (blue line), observed 5th and 95th percentiles (dashed red lines), predicted median (black line) and 90% prediction interval (shaded area) covering the predicted 5th and 95th percentiles (Color figure online)

All fixed and stochastic parameters were estimated with good precision (RSE < 35%, Table 4). The eta shrinkages were 8, 9 and 18% for IIV on CL, sVAP-1_c_ and SL, respectively, while the epsilon shrinkages were 3, 6 and 3% for ASP8232 concentration, sVAP-1 concentration and VAP-1 activity, respectively. Correlations between structural parameter estimates were between − 0.95 and 0.95, with the strongest correlation, 0.91, observed between the K_D_ and power of the VAP-1 concentration—activity relationship. The NONMEM model code is available in the Supplementary Material: in this code, sVAP-1_c_, mVAP-1_c_, mVAP-1_p1_ and mVAP-1_p2_ are referred to as Bmax, Bmax2, Bmax3 and Bmax4, respectively. Overall, the ASP8232 PK-PD model is able to describe the observed ASP8232 and sVAP-1 plasma concentrations and VAP-1 plasma activities well across studies. For the ALBUM study, the VPCs show that the model is able to adequately describe the central tendency and variability in the data for the ASP8232 and sVAP-1 plasma concentrations and the plasma VAP-1 activity (Figs. 3–5). For the three other clinical studies, the VPCs for ASP8232 and sVAP-1 plasma concentrations and VAP-1 plasma activities are presented in the Supplementary Material.

**Table 4:** Parameter estimates of the ASP8232 PK-PD TMDD model

Parameter (unit)	Value	RSE (%)	LLCI	ULCI
k_a_ (1/h)	3.12	12.3	2.37	3.88
LAG (h)	0.311	8.39	0.26	0.362
CL/F1 (L/h)	17.6	4.52	16	19.1
V2/F1 (L)	210	7.76	178	242
Q/F1 (L/h)	37.6	12.1	28.7	46.5
V3/V2 (-)	1^a,b^	–	–	–
Q2/F1 (L/h)	80.5	15.5	56.1	105
V4/F1 (L)	26.7	17.4	17.6	35.8
Emax eGFR on CL/F1	1.3	15.5	0.905	1.69
EC_50_ eGFR on CL/F1 (mL/min/1.73m^2^)	77	3.95	71	83
POW eGFR on CL/F1	10^a^	–	–	–
sVAP1_c_ (nM)	5.52	1.97	5.3	5.73
Factor for mVAP1_c_	2.13^c^	15.9	1.47	2.79
Factor for mVAP1_p1_	52^c^	11	40.8	63.2
Factor for mVAP1_p2_	1^a,d^	–	–	–
K_D_ (nM)	0.929	8.18	0.78	1.08
SL (1/h)	851	2.99	802	901
POW	0.851	1.76	0.822	0.881
Factor res error phase 2 studies	1.88	10.4	1.49	2.26
eGFR on F1	− 0.257	32.8	− 0.422	− 0.0919
SEX on VAP-1 concentrations	0.125	28.4	0.0553	0.195
ω^2^_CL/F1_	0.128	15.4	0.0896	0.167
ω_CL/F1,sVAP1c_	0.0213	42.4	0.00361	0.039
ω^2^_sVAP1c_	0.0735	8.59	0.0611	0.0859
ω_CL/F1,SL_	− 0.0301	29.3	− 0.0474	− 0.0128
ω_sVAP1c,SL_	− 0.0222	23.7	− 0.0325	− 0.0119
ω^2^_SL_	0.0574	13.7	0.042	0.0727
σ^2^_log(PK)_^e^	0.115	8.33	0.0959	0.133
σ^2^_log(VAP-1 concentration)_^e^	0.0351	5.92	0.0311	0.0392
σ^2^_log(VAP-1 activity)_^e^	0.0696	8.67	0.0578	0.0815

^a^Fixed

^b^Factor: V3 = 1 × V2

^c^Factor: mVAP1_c_ = 2.13 × sVAP1_c_ and mVAP1_p1_ = 52 × sVAP1_c_

^d^Factor: mVAP1_p2_ = 1 × mVAP1_p1_

^e^additive error on log scale

*RSE* Relative Standard Error , *L/ULCI* Lower/Upper Limit of 95th Confidence Interval

**Fig. 6:**
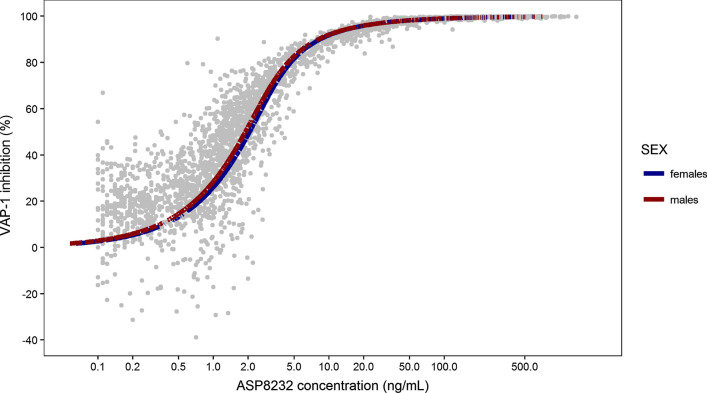
ASP8232 plasma concentration—VAP-1 inhibition effect relationship. Observations (grey dots) and population prediction of the model for males (red line) or females (blue line). The observed VAP-1 inhibition values were obtained as described in Eq. 13. Negative VAP-1 inhibition observations result from measurement error (Color figure online)

The concentration-effect relationship (ASP8232 plasma concentration-VAP-1 percent inhibition) shows that, as observed in the data, the model predicts a near-complete VAP-1 inhibition for ASP8232 concentrations > 50 ng/mL, while below 0.2 ng/mL the inhibition is expected to be minimal (Fig. 6). 50% VAP-1 inhibition is expected at an ASP8232 concentration of approximately 2 ng/mL, irrespective of sex. Below 0.5 ng/mL, there appears to be a bias toward a lower population prediction for VAP-1 inhibition compared with the observed data, as the model predicts that the VAP-1 inhibition should tend to zero with decreasing ASP8232 concentration, while from the observed data, VAP-1 inhibition remains at approximately 20%, even at the lowest measured concentrations.

### Model simulation

**Fig. 7:**
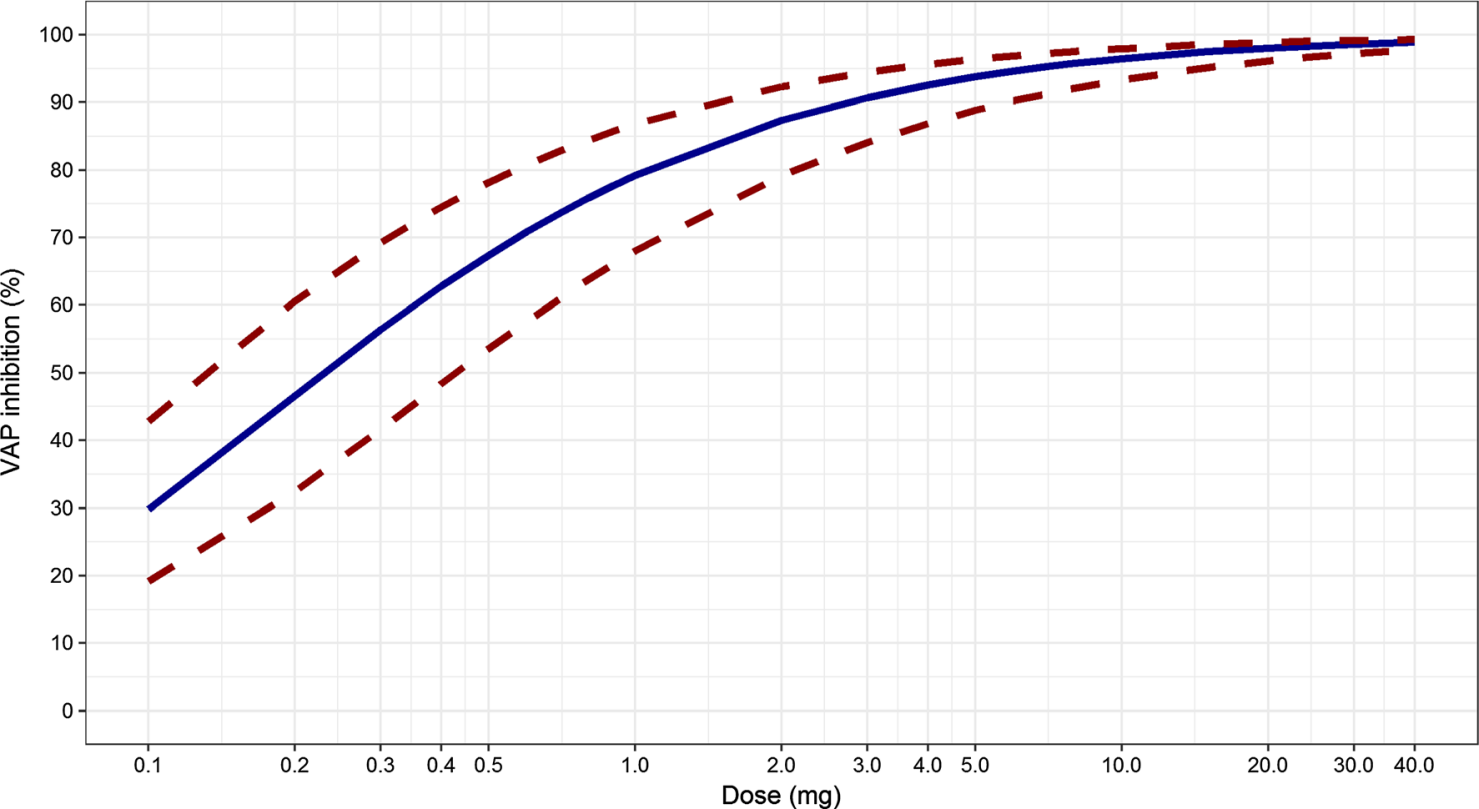
Model-predicted average 24 h inhibition of VAP-1 activity (%) versus ASP8232 dose after 52 weeks of daily oral dosing for DKD patients. Simulation based on individual predictions; Median (blue), 5th and 95th percentile (dashed red) of 10,000 replicates (Color figure online)

**Fig. 8:**
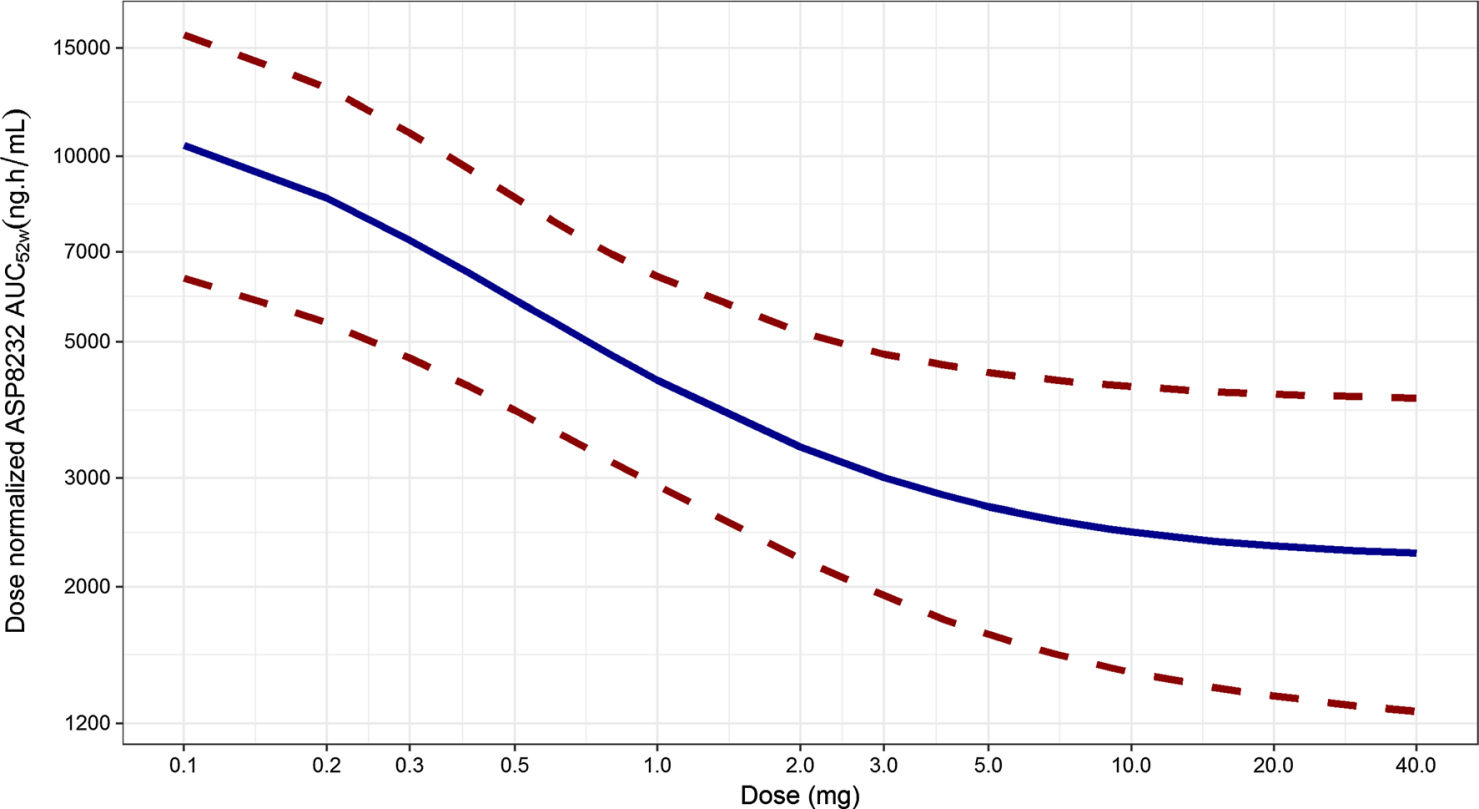
Model-predicted dose-normalized ASP8232 AUC_24h,52w_, normalized to a dose of 40 mg, versus ASP8232 dose after 52 weeks of daily oral dosing for DKD patients. Simulation based on individual predictions; Median (blue), 5th and 95th percentile (dashed red) of 10,000 replicates. Dose-normalized AUC’s, with 40 mg qd dosing as reference, were obtained via 40*AUC/Dose (Color figure online)

**Fig. 9:**
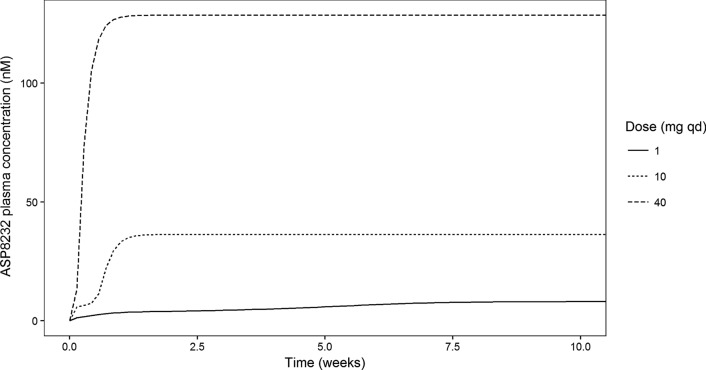
Simulation of ASP8232 plasma trough concentration (nM) over time for a typical subject receiving 1, 10 or 40 mg daily ASP8232 doses. Typical subject was defined as a male subject with eGFR of 44 mL/min/1.73m^2^, i.e. the median value observed in the ALBUM study. Simulated trough concentrations were plotted

**Fig. 10:**
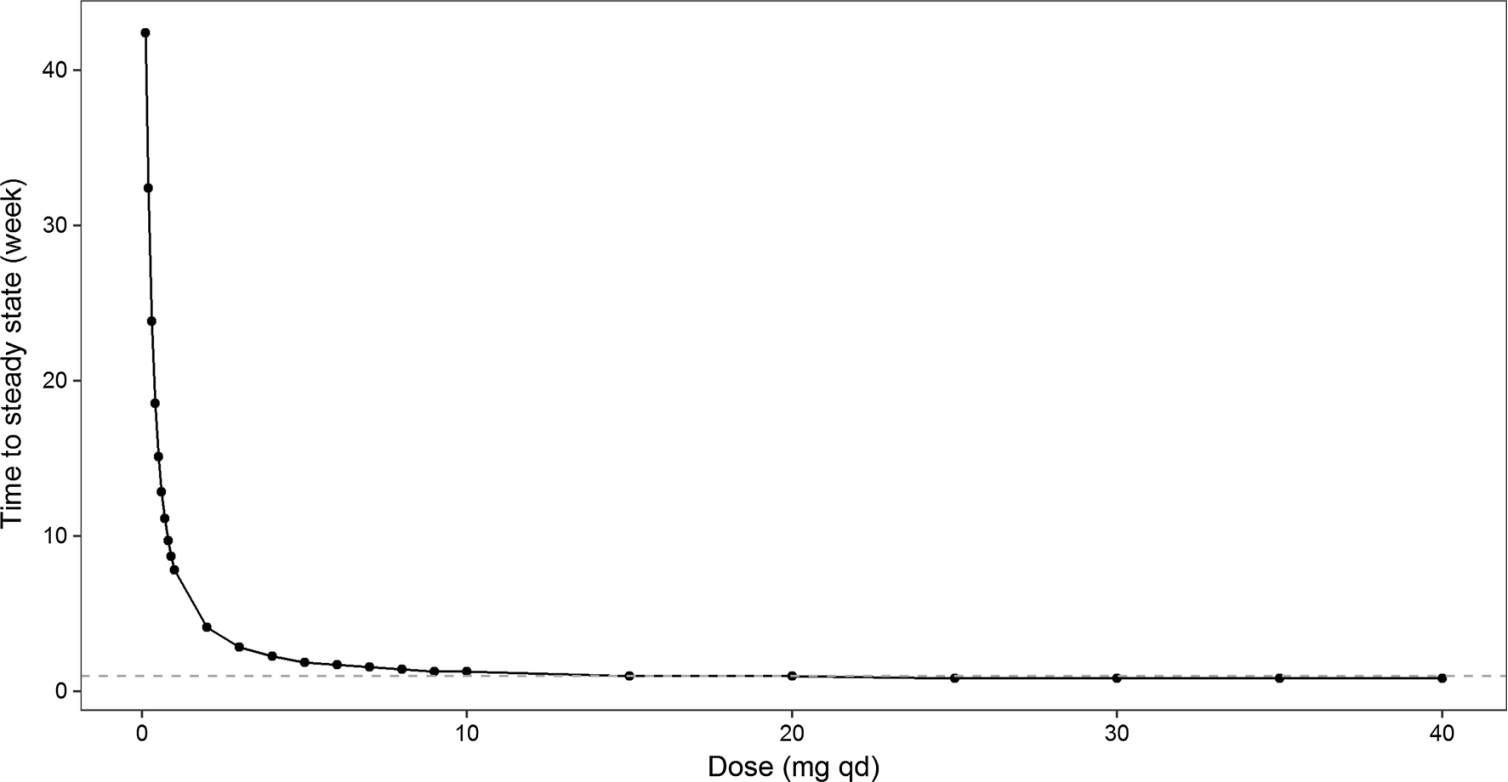
Simulation of the time to reach steady state for ASP8232 plasma concentration or a typical subject receiving a range of daily ASP8232 doses between 0.1 and 40 mg. Time to steady state was defined as the time to reach 97% of the concentration predicted after 52 weeks of daily ASP8232 dosing. Typical subject was defined as a male subject with eGFR of 44 mL/min/1.73m^2^, i.e. the median value observed in the ALBUM study. Dashed line at 1 week for clarity

Simulations were conducted for DKD patients given between 0.1 and 40 mg of ASP8232 daily for 52 weeks. The median, 5th and 95th percentiles of the simulations are shown in Fig. 7. On average, the inhibition of VAP-1 is expected to be above 90% for ≥ 3 mg daily doses, while inhibition is expected to drop below 50% for < 0.3 mg daily doses. The larger between subject variability in response for doses < 1 mg as compared to higher doses, is expected, as VAP-1 inhibition cannot exceed the maximum of 100%. Similar simulations were conducted to describe the relationship between dose and dose normalized AUC_24h,52w_. Due to VAP-1 binding, ASP8232 is subjected to target mediated drug elimination, and thus the ASP8232 pharmacokinetics are non-linear. The impact of this non-linearity was visualized by dose-normalizing with 40 mg as reference, whereby the exposure increases less than proportional with dose, and whereby doses ≥ 30 mg result in similar dose normalized exposure as 40 mg (Fig. 8). Compared with 40 mg daily dosing, the dose-normalized exposure is expected to be approximately fourfold higher than daily dosing with 0.1 mg for 52 weeks. In addition, this non-linearity is apparent from the typical individual simulations predicting the ASP8232 concentration – time profile, and the corresponding key secondary PK parameters, for a range of daily ASP8232 doses (Fig. 9, Table 5 and Supplementary Figure S16). From these simulations, the time to reach steady state for ASP8232 concentrations is predicted to be one week for daily doses ≥ 10 mg, and higher for lower doses, e.g. > 40 weeks for 0.1 mg daily dosing (Fig. 10 and Table 5).

**Table 5:** Secondary ASP8232 PK parameters for a typical subject

Dose (mg)	AUC_24h,52w_ (ng*h/mL)	Cmax_52w_ (ng/mL)	Tmax_52w_ (h)	Tss (weeks)	Apparent t1/2 (weeks)
0.1	24.4	1.1	2	42.4	8.5
1	101.8	6.2	1.5	7.9	1.6
5	337.8	25.1	1.6	1.9	0.4
10	622.2	48.1	1.6	1.3	0.3
20	1187.2	94.1	1.6	1	0.2
30	1777.8	140	1.6	0.9	0.2
40	2355.6	186	1.6	0.9	0.2

## Discussion

The PK and PD of ASP8232 were successfully characterized using a TMDD model based on data from healthy volunteers, subjects with renal impairment, patients with DKD, and patients with DME. Differences between these populations could be fully explained by an eGFR effect on CL and F1, and no additional descriptive population effects had to be included. Renal excretion of ASP8232 is a minor part of the total elimination of ASP8232. An effect of eGFR exposure (CL and F1) might be explained by the presence of uremic toxins that inhibit pre-systemic metabolizing enzymes in subjects with decreased renal function [27, 28]. Subsequently, the resulting relationship between exposure and eGFR was visualized (Fig. 2), showing that on average, the AUC_24h,52w_ is expected to be 3.3 fold higher for a typical subject with baseline eGFR of 20 versus 110 mL/min/1.73m^2^. Females were found to have 12.5% higher VAP-1 concentrations. However, the impact of this effect appeared to be minimal in terms of AUC_24h,52w_ and VAP-1 inhibition (Figs. 2 and 6, respectively).

The developed TMDD model relies on several assumptions. A critical assumption was that of binding equilibrium, i.e. compared with other processes, binding of ASP8232 to VAP-1 was assumed to be rapid, while VAP-1 turnover and elimination of the VAP-1-ASP8232 complex were assumed to be negligible. As such, although mechanistically expected, there was no target turnover or elimination of the complex implemented in the model. This model limitation was deemed acceptable, given that the TMDD model was able to successfully describe the observed ASP8232 and sVAP-1 plasma concentrations, as well as the VAP-1 plasma activities across all studies. Thus, the need for a more complex model was not pursued in favor of the more parsimonious alternative. However, caution is advised when interpreting simulations beyond the available dose range in the clinical studies, as VAP-1 turnover might be relevant, and thus its exclusion could lead to some bias in the estimated parameters. Another assumption was that the measured VAP-1 plasma activity was driven by the unbound sVAP-1 plasma concentrations and this relationship was assumed to be constant over time. The placebo data support the assumption that the VAP-1 plasma concentration-activity relationship was constant over time as both the VAP-1 plasma concentration and activity did not change over time.

The model was able to adequately describe the data for all studies. However, below ASP8232 concentrations of 0.5 ng/mL, a discrepancy between model predictions and observations was seen for VAP-1 activity expressed as percent inhibition of activity (Fig. 6). It was a conscious decision to stick with a model without inhibition of VAP-1 activity in the absence of ASP8232 exposure, despite the discrepancies with the observed data. This model was considered more mechanistically plausible, and the observed higher VAP-1 activity was thought to be non-drug related. One possible explanation could be that the baseline VAP-1 observation was lower than the actual endogenous level, which influences the observed, and to a lesser extent, the model-predicted VAP-1 inhibition. This hypothesis is supported by higher measurements on day 1 as compared to the baseline observation. Another explanation could be a variation in VAP-1 activity over time, which was seen in the placebo groups of study 8232-CL-0001.

Nonetheless, at higher concentrations, the model adequately predicts the increase in VAP-1 inhibition with ASP8232 concentration. The model was therefore used to predict the VAP-1 inhibition for dose levels not currently evaluated in the clinic to aid drug development, for example for selecting the doses to be included in a Phase 2 dose-finding study. Population simulations were performed predicting the expected PD response in DKD patients upon 1-year treatment with 0.1 to 40 mg daily ASP8232 (Fig. 6). The results indicate that a low effect response (defined as below 50% VAP-1 inhibition) would be obtained on average in DKD patients by ASP8232 dosing below 0.3 mg, while a high response (above 90% VAP-1 inhibition) would be achieved by doses above 3 mg.

In conclusion, the PK-PD of ASP8232 was successfully characterized using a TMDD model. This model provides a robust tool to simulate plasma VAP-1 activity in relation to drug exposure and may be used to guide dose selection in future clinical trials with ASP8232.

## Electronic supplementary material

Below is the link to the electronic supplementary material.

Supplementary file1 (TXT 3 kb)

Supplementary Figure 2: Visual predictive check of ASP8232 plasma concentration in the singledose cohort of study 8232-CL-0001. (PNG 35 kb)

Supplementary Figure 2: Visual predictive check of ASP8232 plasma concentration in themultiple dose cohort of study 8232-CL-0001. (PNG 35 kb)

Supplementary Figure 3: Visual predictive check of ASP8232 plasma concentration in the singledose cohort of study 8232-CL-0002. (PNG 36 kb)

Supplementary Figure 4: Visual predictive check of ASP8232 plasma concentration in themultiple dose cohort of study 8232-CL-0002. (PNG 44 kb)

Supplementary Figure 5: Visual predictive check of ASP8232 plasma concentration in study8232-CL-3001. (PNG 37 kb)

Supplementary Figure 6: Visual predictive check of VAP-1 plasma concentration in study 8232-CL-3001. (PNG 37 kb)

Supplementary Figure 7: Visual predictive check of VAP-1 plasma activity in the single dosecohort of study 8232-CL-0001. (PNG 29 kb)

Supplementary Figure 8: Visual predictive check of VAP-1 plasma activity in the multiple dosecohort of study 8232-CL-0001. (PNG 34 kb)

Supplementary Figure 9: Visual predictive check of VAP-1 plasma activity in the single dosecohort of study 8232-CL-0002. (PNG 32 kb)

Supplementary Figure 10: Visual predictive check of VAP-1 plasma activity in the multiple dosecohort of study 8232-CL-0002. (PNG 33 kb)

Supplementary Figure 11: Visual predictive check of VAP-1 plasma activity in study 8232-CL-3001. (PNG 36 kb)

Supplementary Figure 12: Visual predictive check of ASP8232 plasma concentration up to 50hafter dose in the single dose cohort of study 8232-CL-0001. (PNG 36 kb)

Supplementary Figure 13: Visual predictive check of ASP8232 plasma concentration up to 50hafter dose in the multiple dose cohort of study 8232-CL-0001. (PNG 29 kb)

Supplementary Figure 14: Visual predictive check of VAP-1 plasma activity up to 50h afterdose in the single dose cohort of study 8232-CL-0001. (PNG 31 kb)

Supplementary Figure 14: Visual predictive check of VAP-1 plasma activity up to 50h afterdose in the single dose cohort of study 8232-CL-0001. (PNG 28 kb)

Supplementary Figure 15: Visual predictive check of VAP-1 plasma activity up to 50h afterdose in the multiple dose cohort of study 8232-CL-0001. (PNG 39 kb)
