# Supplementary figures and images for: Population pharmacokinetics and pharmacodynamics of a novel vascular adhesion protein-1 inhibitor using a multiple-target mediated drug disposition model
Source: J Pharmacokinet Pharmacodyn. 2020 Sep 15;48(1):39–53. doi: 10.1007/s10928-020-09717-w (PMC7979583; doi:10.1007/s10928-020-09717-w)

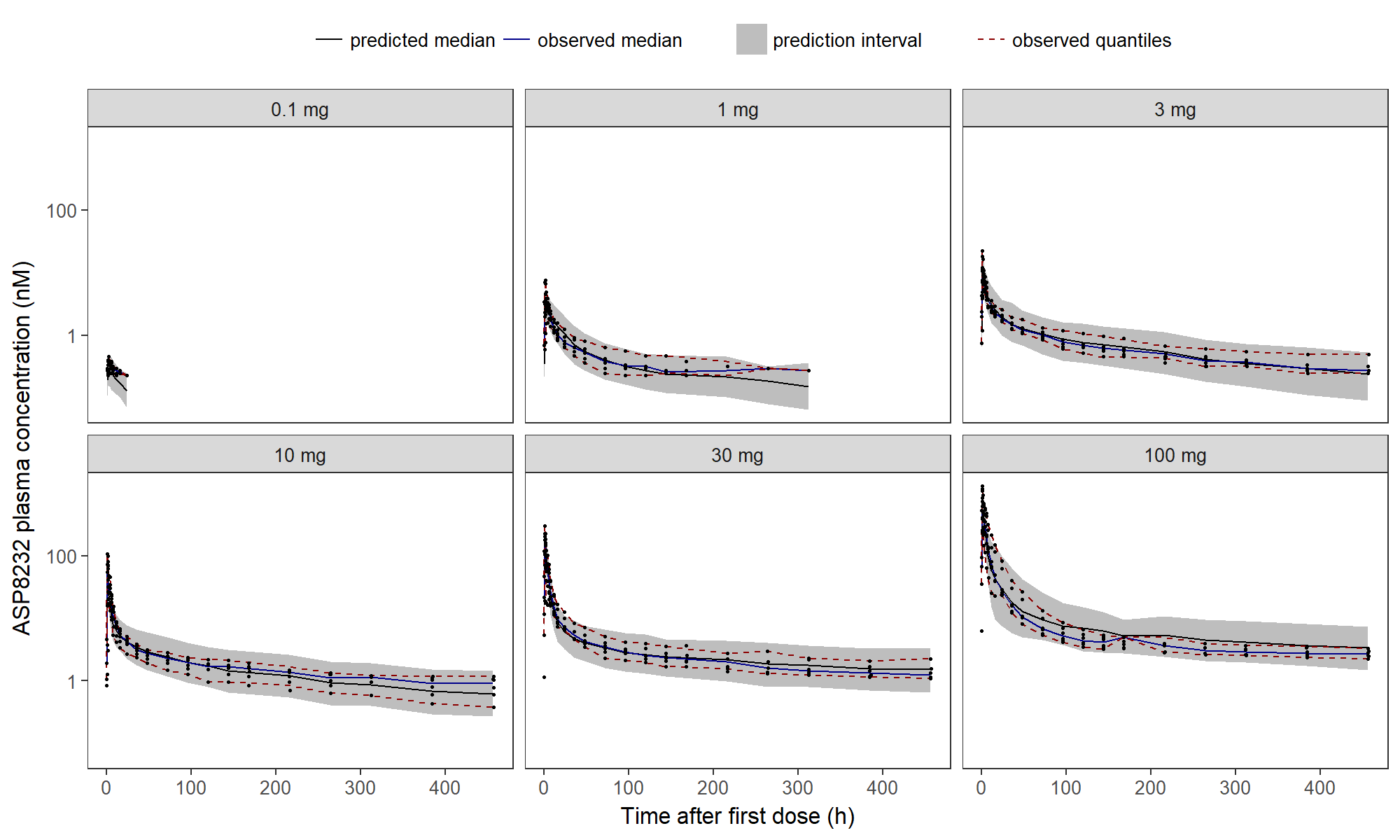

Supplement: Supplementary file 2 — Supplementary Figure 2: Visual predictive check of ASP8232 plasma concentration in the singledose cohort of study 8232-CL-0001. (PNG 35 kb) [file 10928_2020_9717_MOESM2_ESM.png]

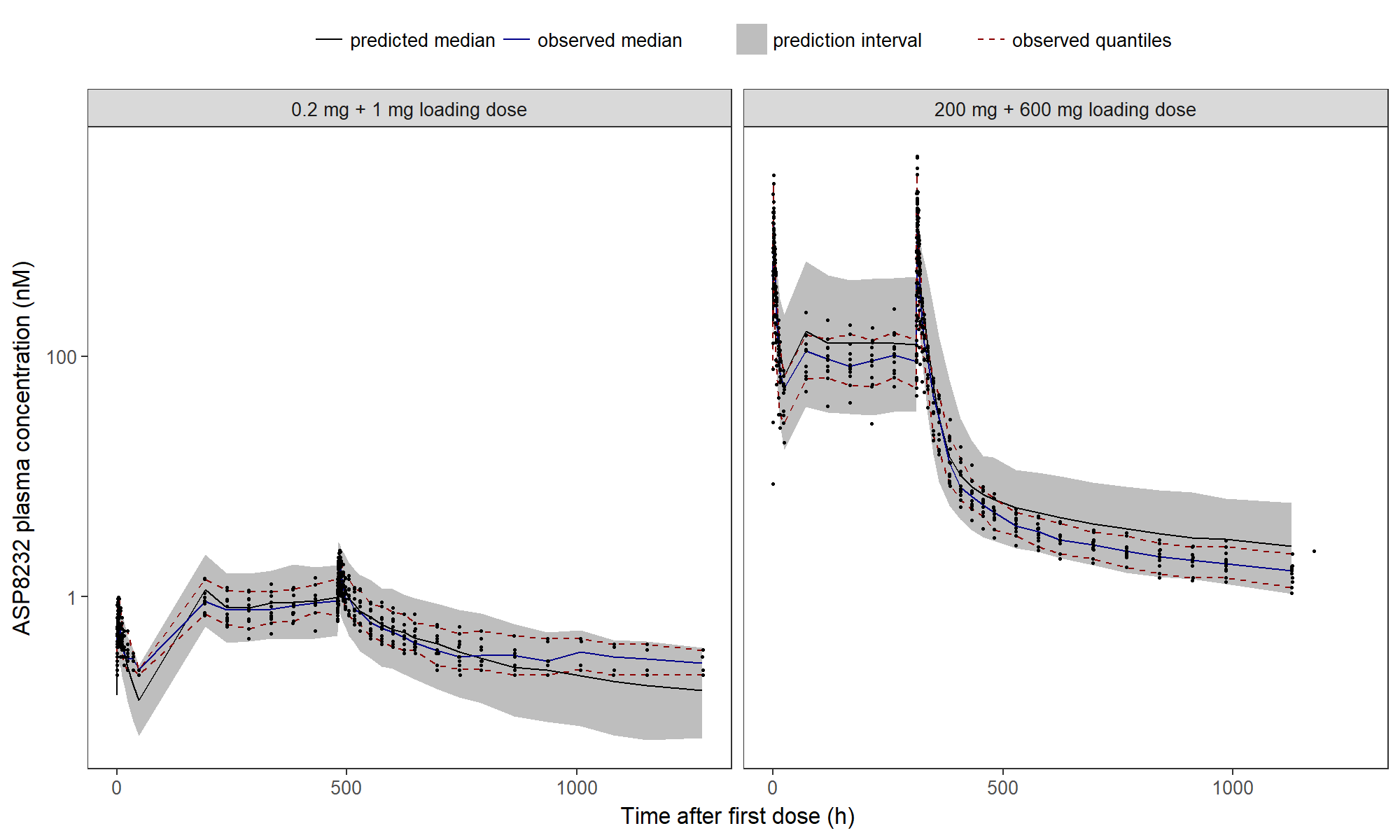

Supplement: Supplementary file 3 — Supplementary Figure 2: Visual predictive check of ASP8232 plasma concentration in themultiple dose cohort of study 8232-CL-0001. (PNG 35 kb) [file 10928_2020_9717_MOESM3_ESM.png]

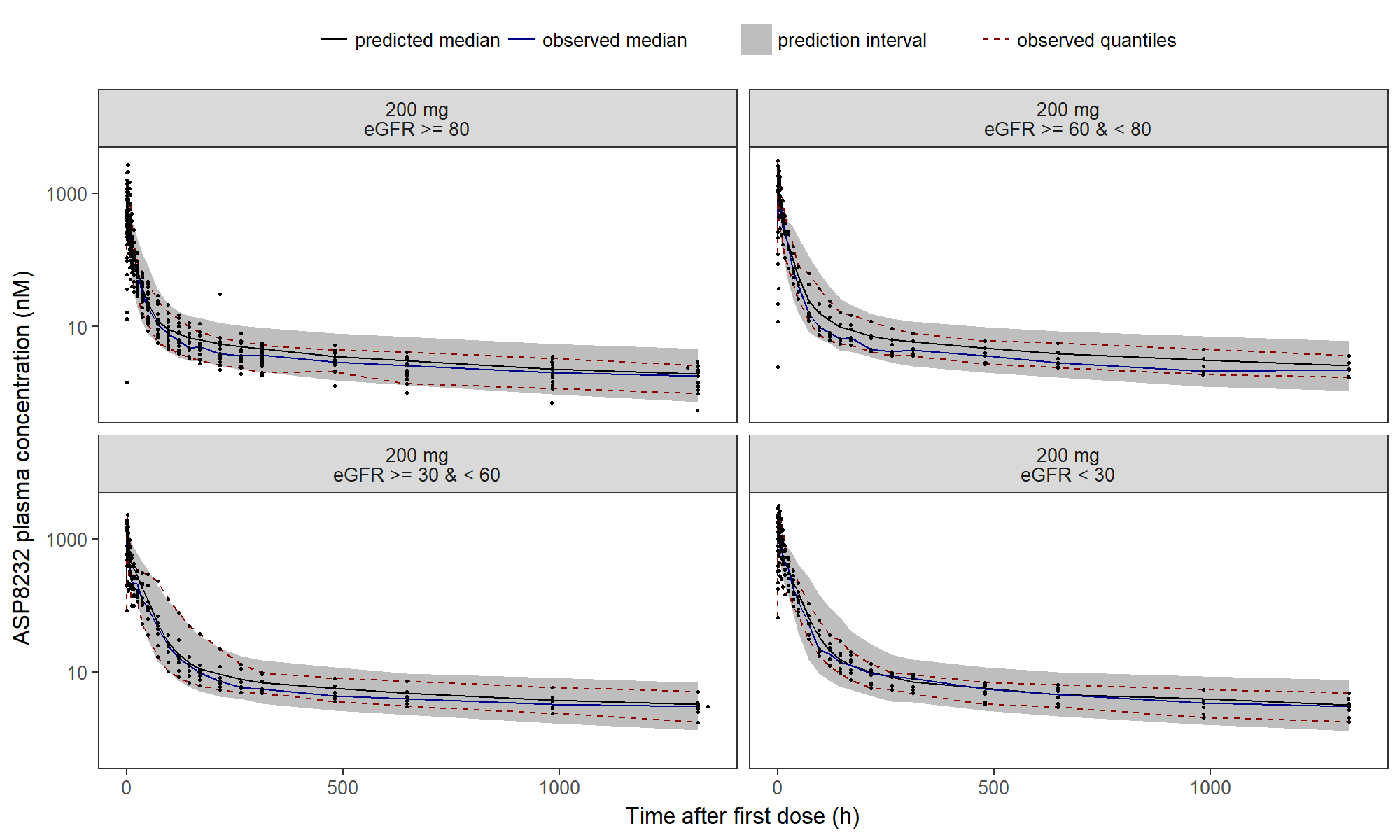

Supplement: Supplementary file 4 — Supplementary Figure 3: Visual predictive check of ASP8232 plasma concentration in the singledose cohort of study 8232-CL-0002. (PNG 36 kb) [file 10928_2020_9717_MOESM4_ESM.png]

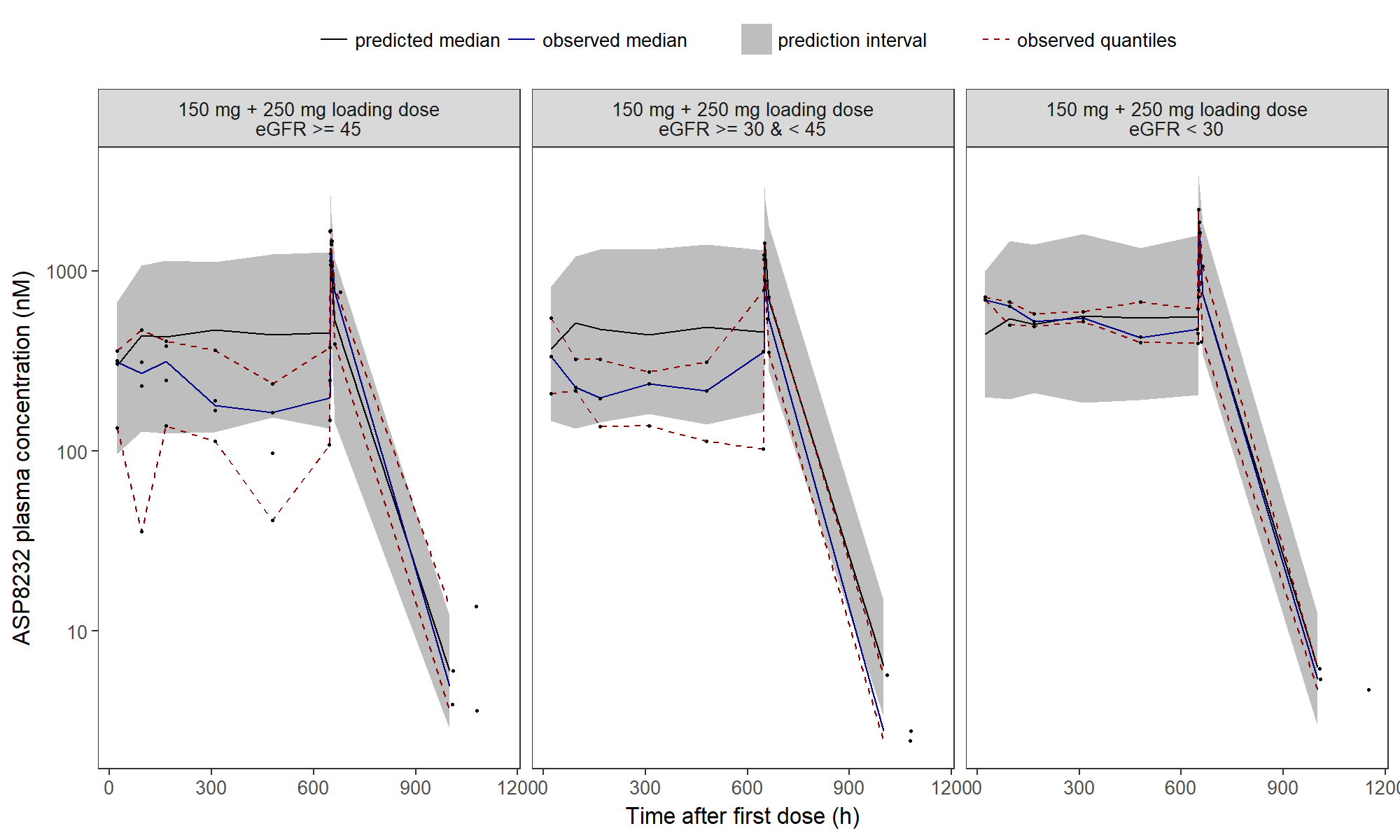

Supplement: Supplementary file 5 — Supplementary Figure 4: Visual predictive check of ASP8232 plasma concentration in themultiple dose cohort of study 8232-CL-0002. (PNG 44 kb) [file 10928_2020_9717_MOESM5_ESM.png]

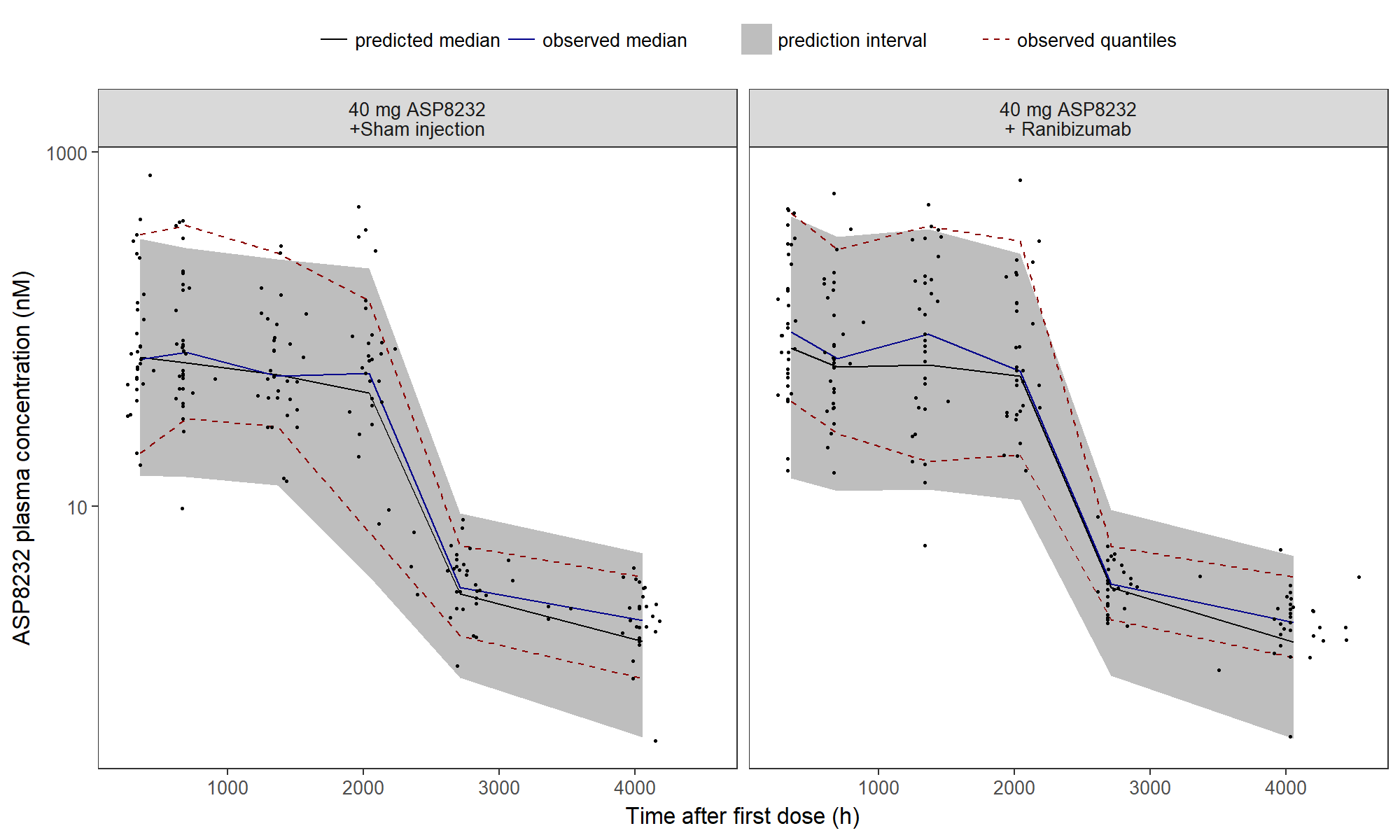

Supplement: Supplementary file 6 — Supplementary Figure 5: Visual predictive check of ASP8232 plasma concentration in study8232-CL-3001. (PNG 37 kb) [file 10928_2020_9717_MOESM6_ESM.png]

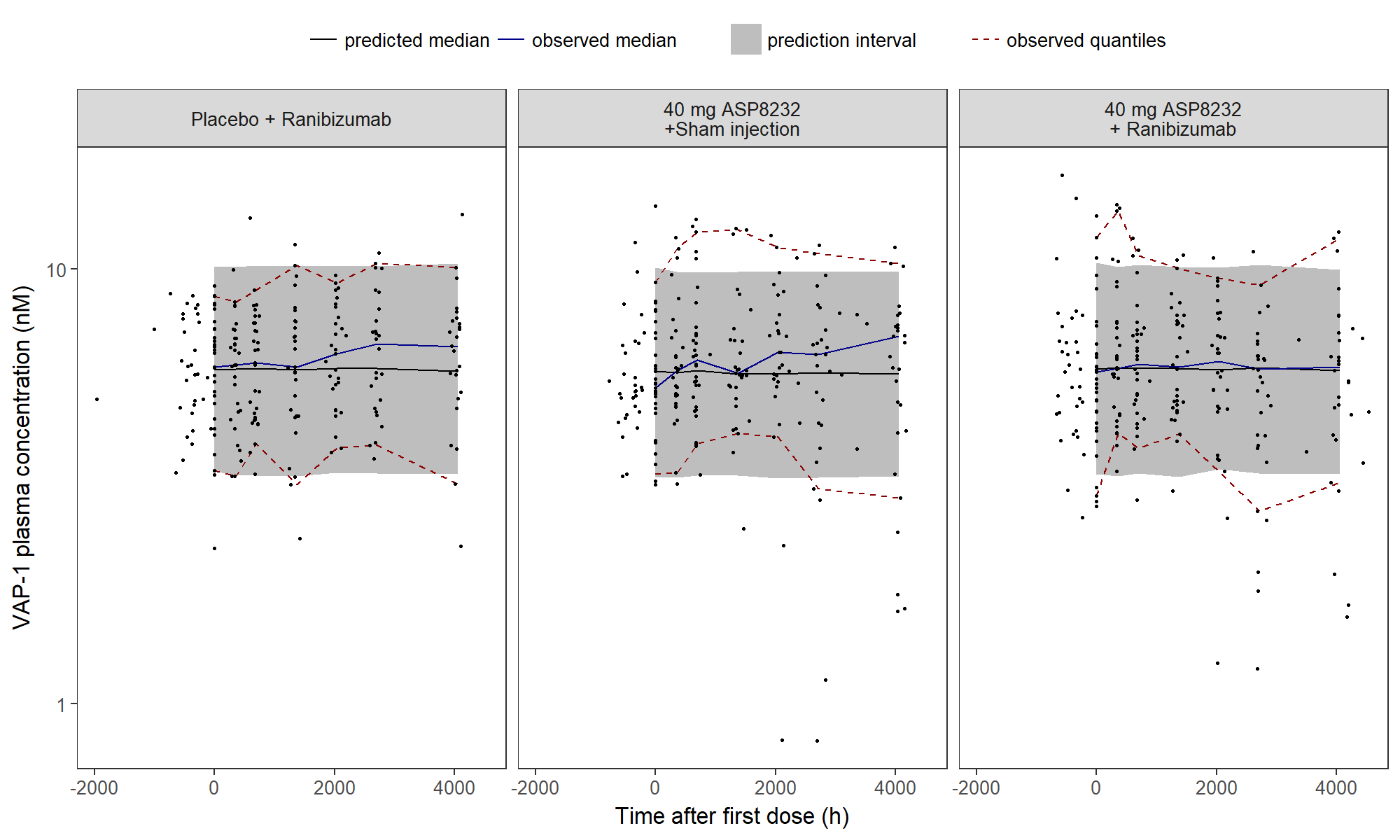

Supplement: Supplementary file 7 — Supplementary Figure 6: Visual predictive check of VAP-1 plasma concentration in study 8232-CL-3001. (PNG 37 kb) [file 10928_2020_9717_MOESM7_ESM.png]

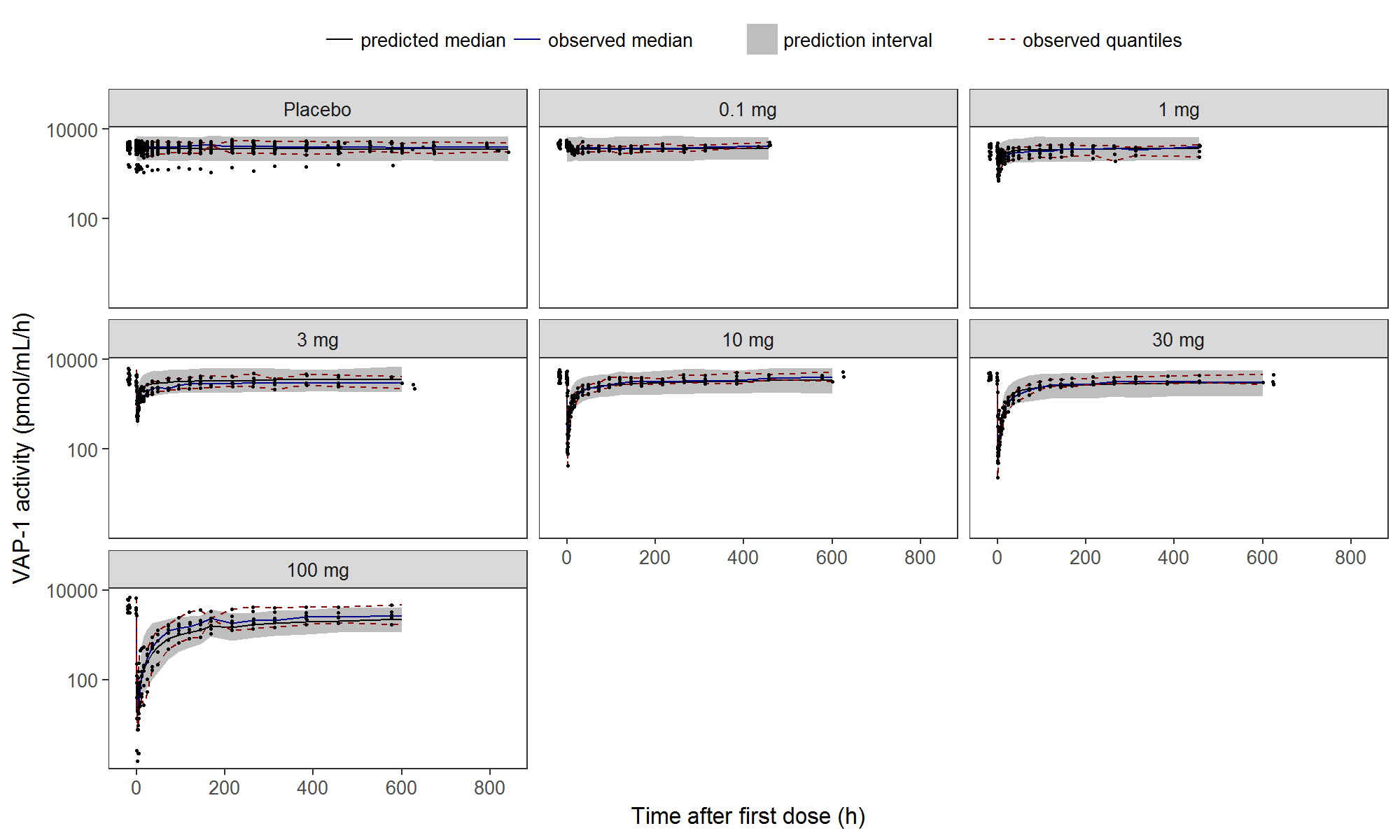

Supplement: Supplementary file 8 — Supplementary Figure 7: Visual predictive check of VAP-1 plasma activity in the single dosecohort of study 8232-CL-0001. (PNG 29 kb) [file 10928_2020_9717_MOESM8_ESM.png]

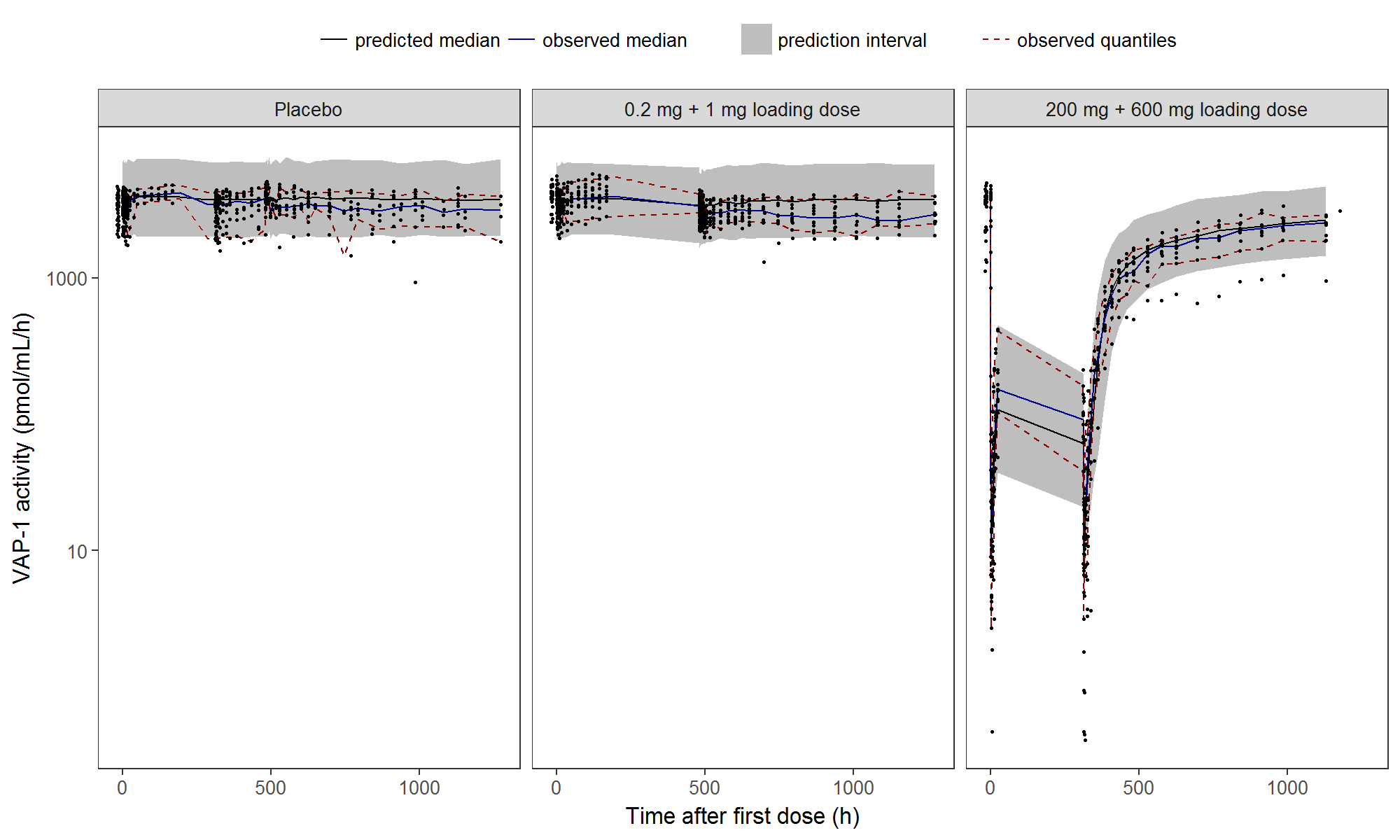

Supplement: Supplementary file 9 — Supplementary Figure 8: Visual predictive check of VAP-1 plasma activity in the multiple dosecohort of study 8232-CL-0001. (PNG 34 kb) [file 10928_2020_9717_MOESM9_ESM.png]

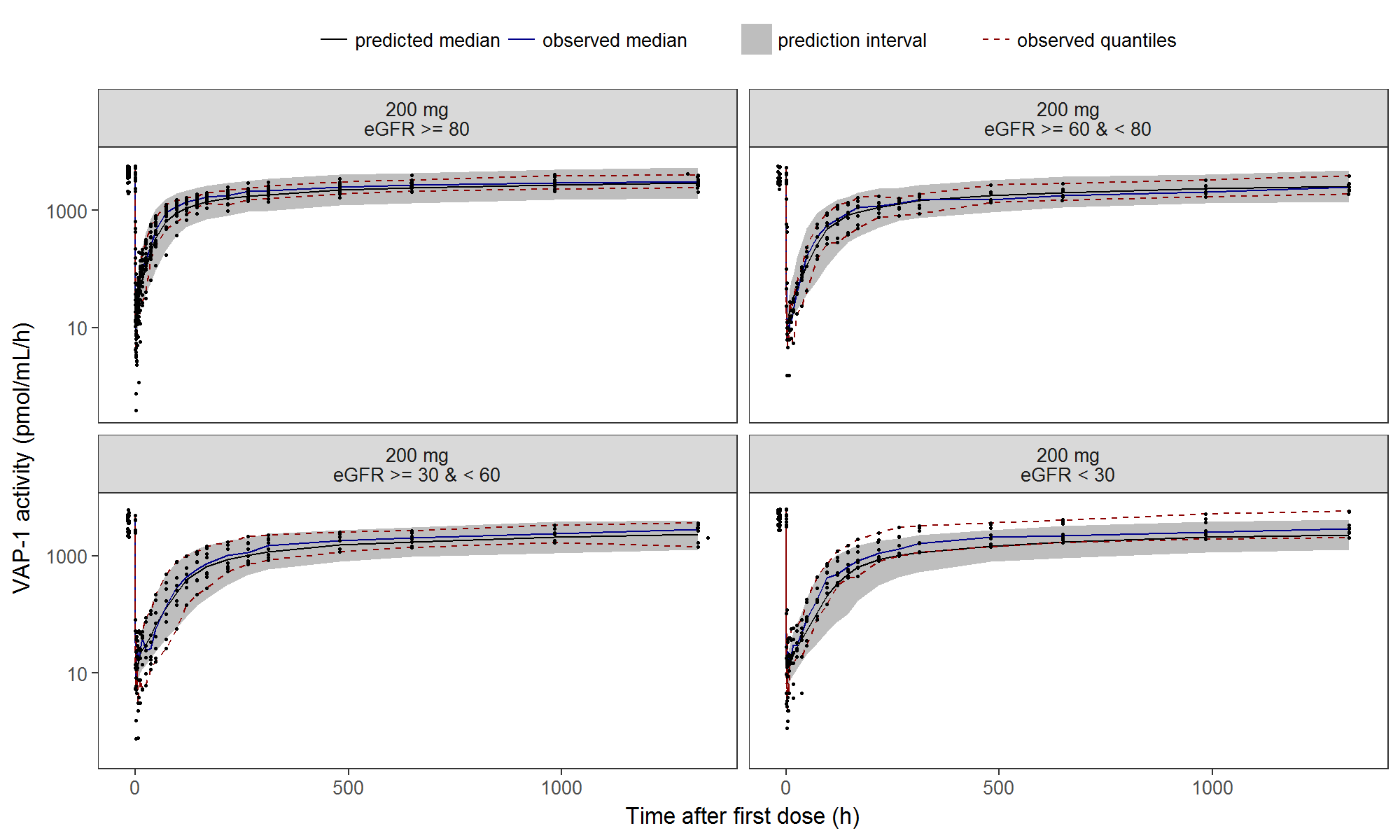

Supplement: Supplementary file 10 — Supplementary Figure 9: Visual predictive check of VAP-1 plasma activity in the single dosecohort of study 8232-CL-0002. (PNG 32 kb) [file 10928_2020_9717_MOESM10_ESM.png]

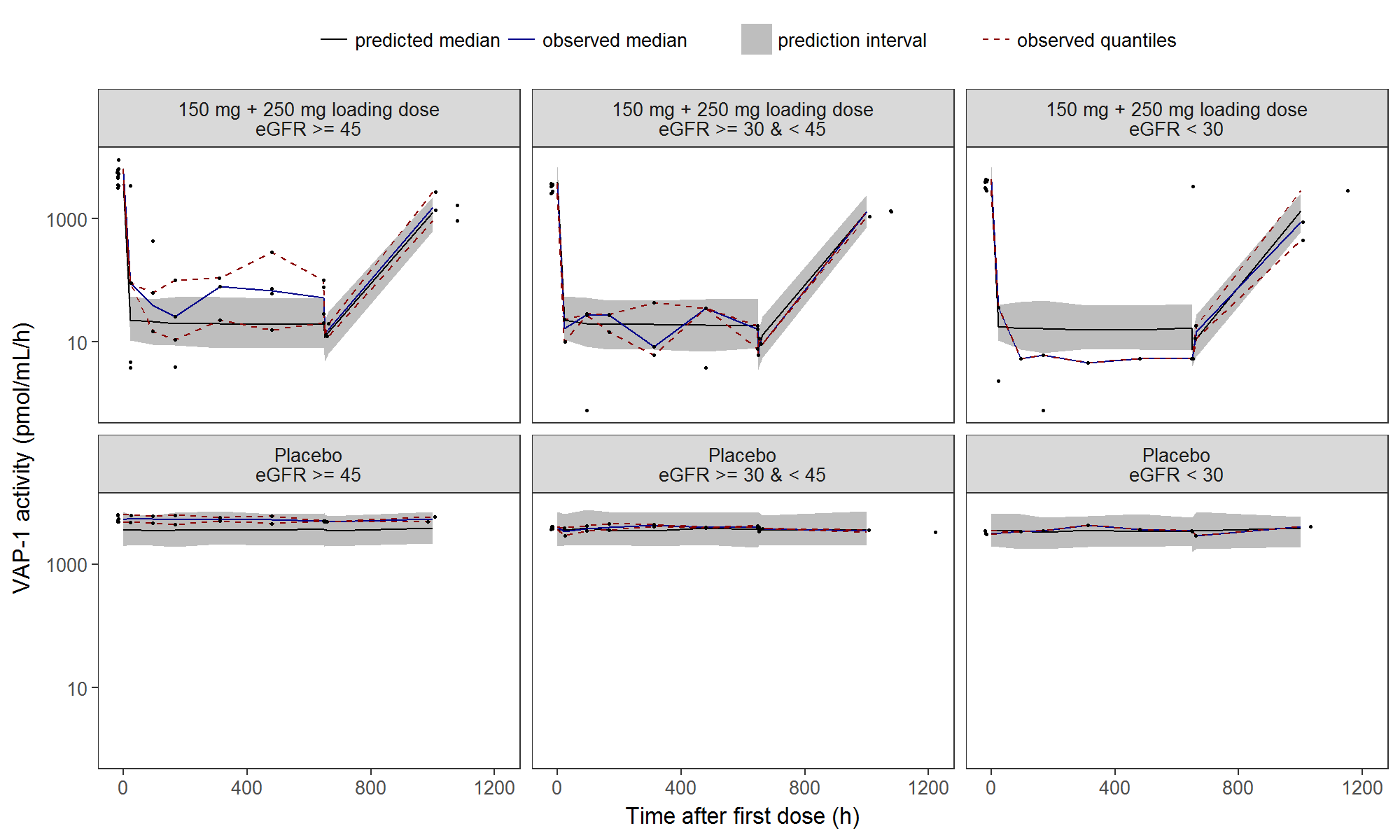

Supplement: Supplementary file 11 — Supplementary Figure 10: Visual predictive check of VAP-1 plasma activity in the multiple dosecohort of study 8232-CL-0002. (PNG 33 kb) [file 10928_2020_9717_MOESM11_ESM.png]

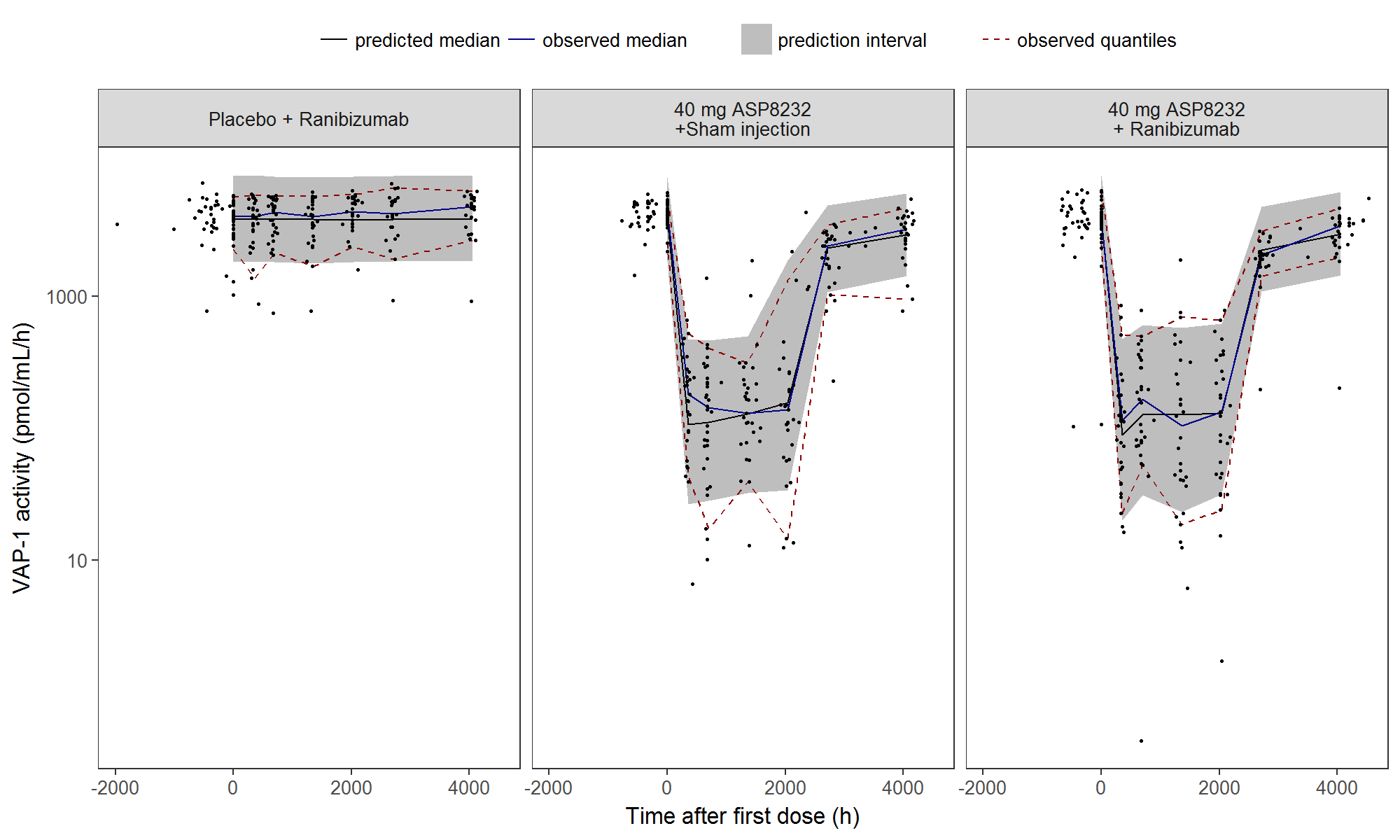

Supplement: Supplementary file 12 — Supplementary Figure 11: Visual predictive check of VAP-1 plasma activity in study 8232-CL-3001. (PNG 36 kb) [file 10928_2020_9717_MOESM12_ESM.png]

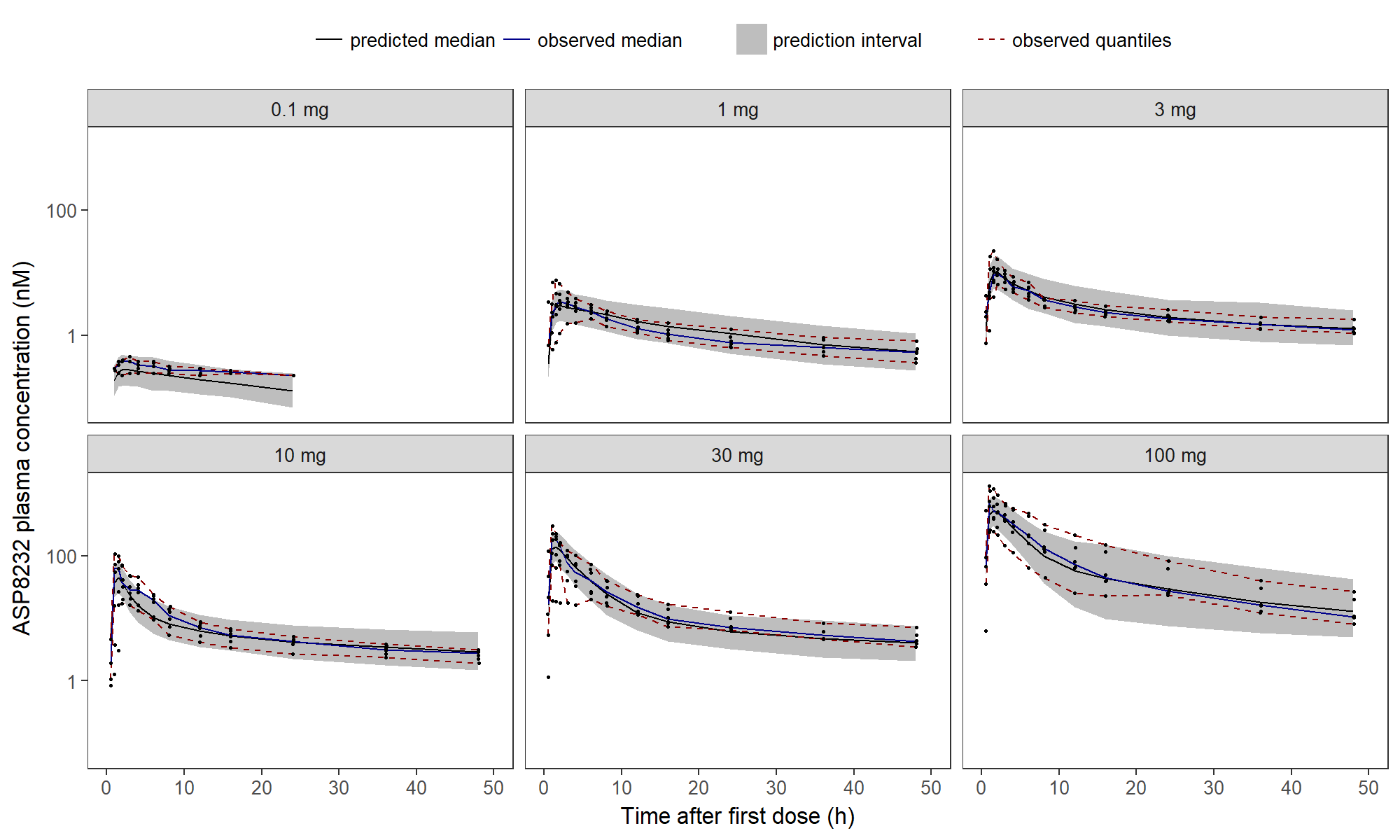

Supplement: Supplementary file 13 — Supplementary Figure 12: Visual predictive check of ASP8232 plasma concentration up to 50hafter dose in the single dose cohort of study 8232-CL-0001. (PNG 36 kb) [file 10928_2020_9717_MOESM13_ESM.png]

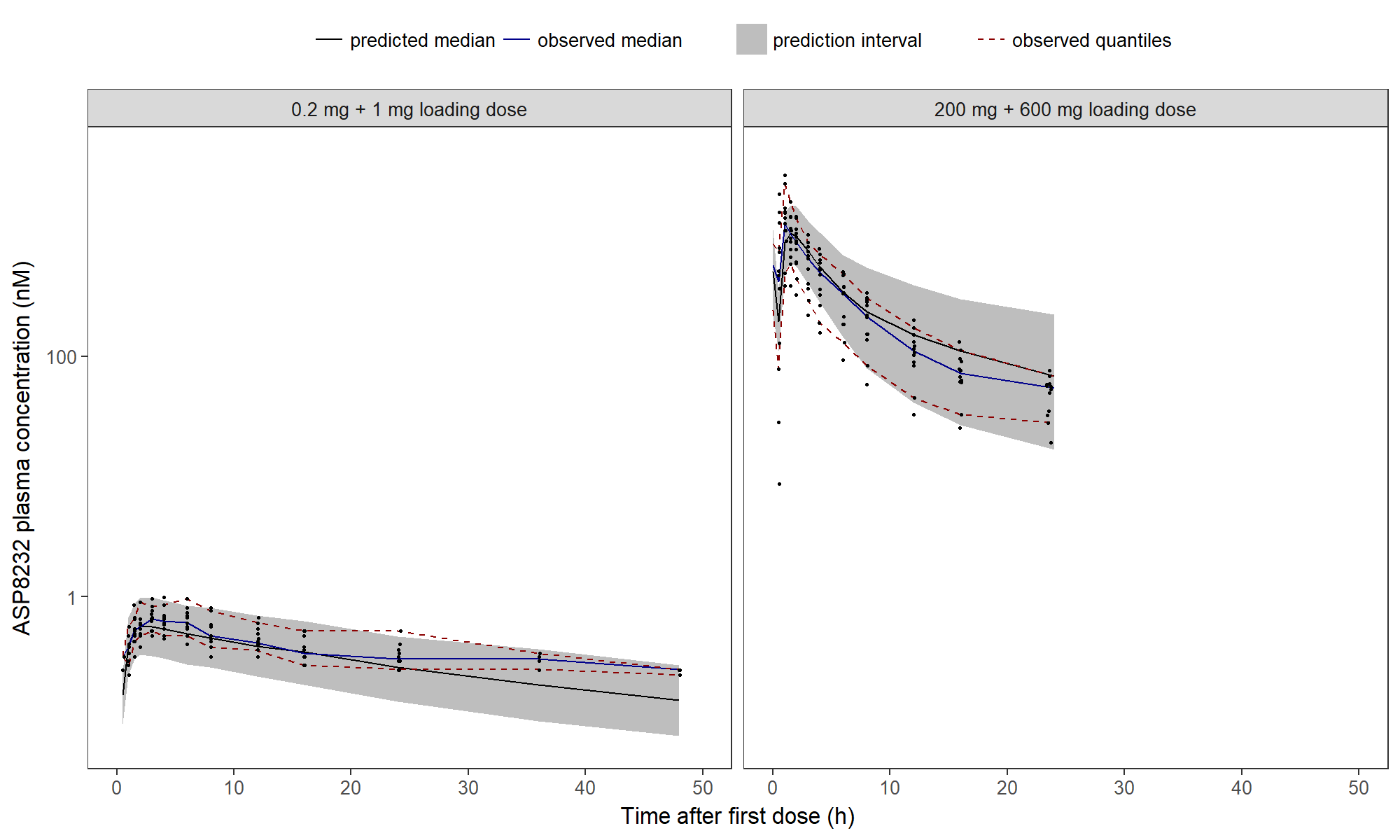

Supplement: Supplementary file 14 — Supplementary Figure 13: Visual predictive check of ASP8232 plasma concentration up to 50hafter dose in the multiple dose cohort of study 8232-CL-0001. (PNG 29 kb) [file 10928_2020_9717_MOESM14_ESM.png]

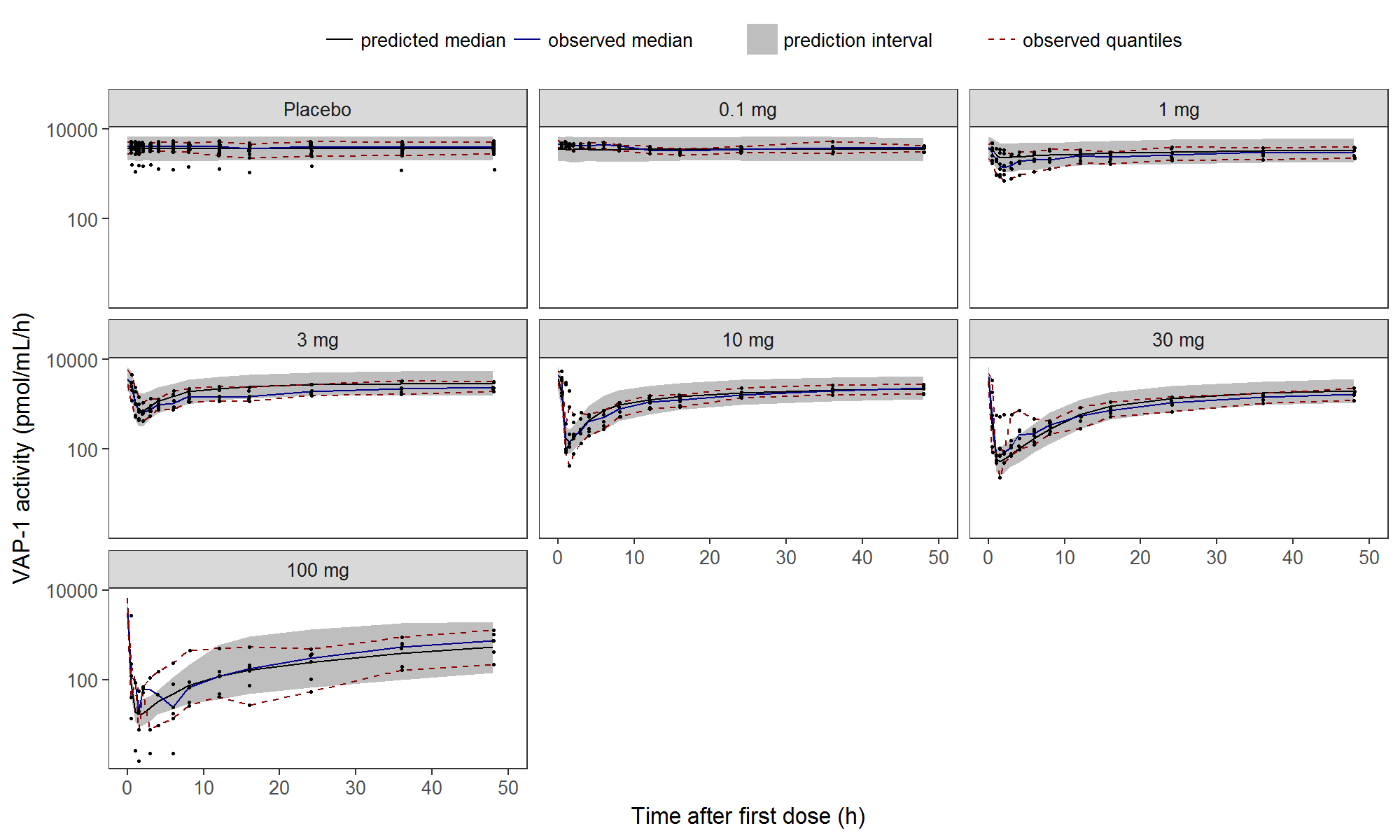

Supplement: Supplementary file 15 — Supplementary Figure 14: Visual predictive check of VAP-1 plasma activity up to 50h afterdose in the single dose cohort of study 8232-CL-0001. (PNG 31 kb) [file 10928_2020_9717_MOESM15_ESM.png]

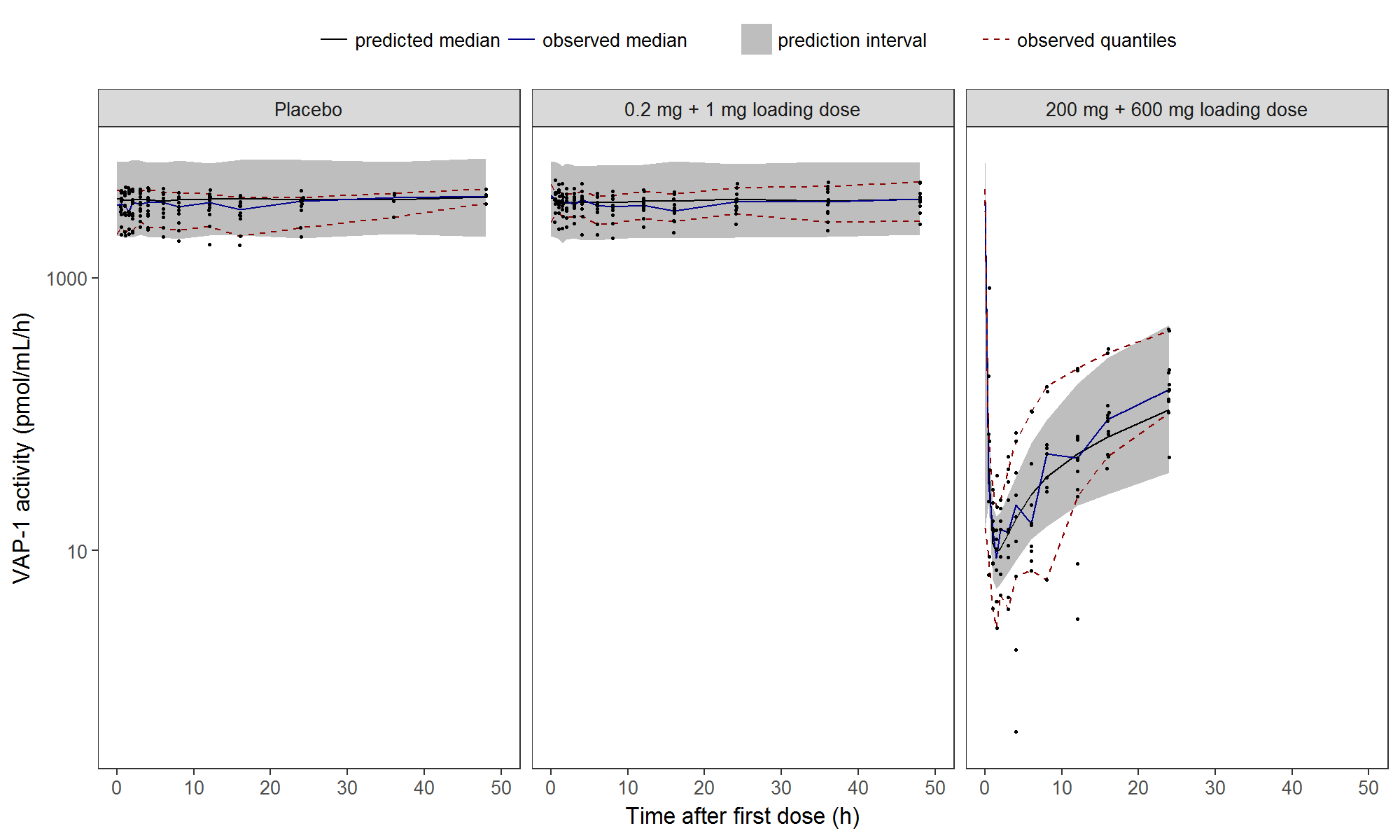

Supplement: Supplementary file 16 — Supplementary Figure 14: Visual predictive check of VAP-1 plasma activity up to 50h afterdose in the single dose cohort of study 8232-CL-0001. (PNG 28 kb) [file 10928_2020_9717_MOESM16_ESM.png]

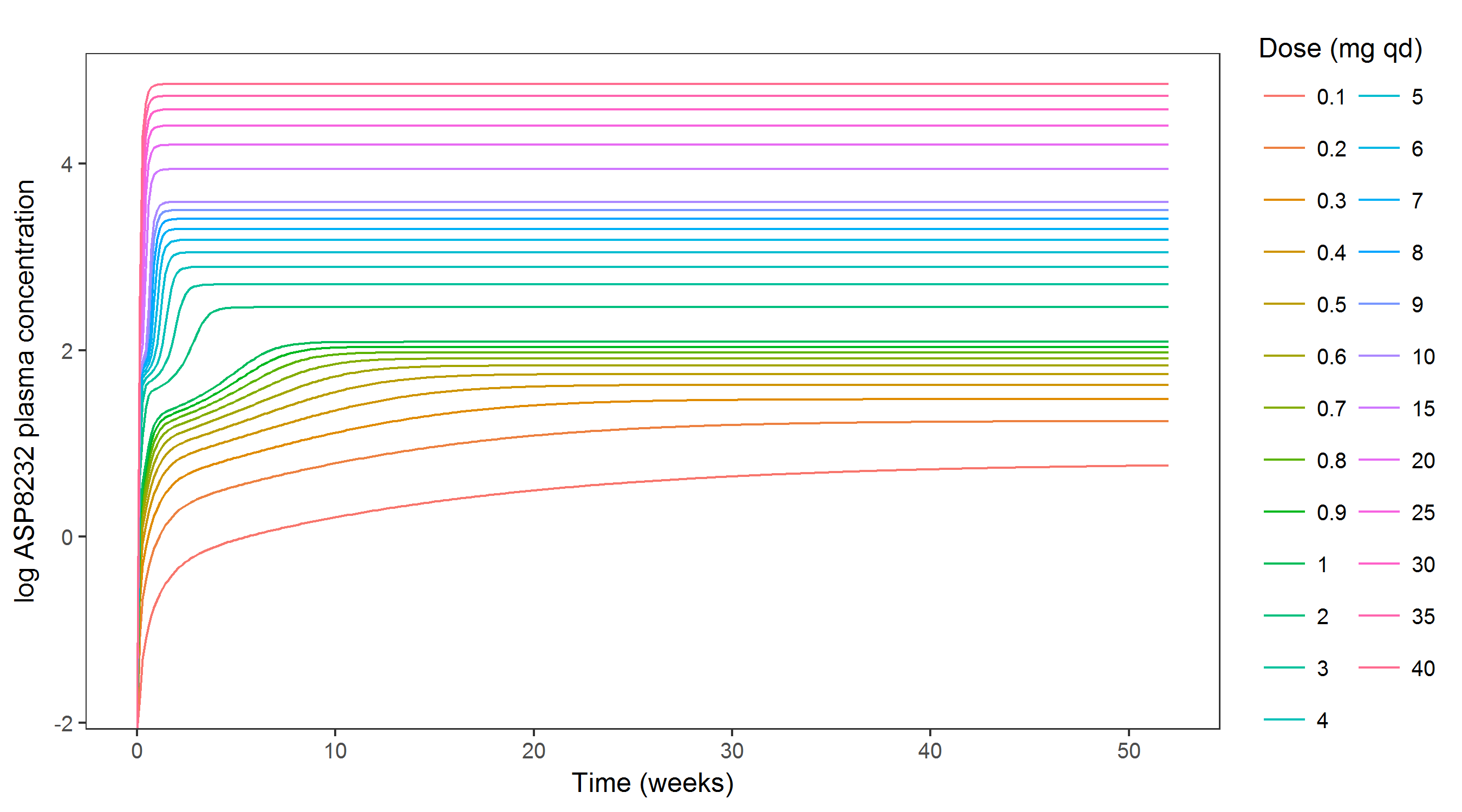

Supplement: Supplementary file 17 — Supplementary Figure 15: Visual predictive check of VAP-1 plasma activity up to 50h afterdose in the multiple dose cohort of study 8232-CL-0001. (PNG 39 kb) [file 10928_2020_9717_MOESM17_ESM.png]
